# Pregabalin Silences Oxaliplatin-Activated Sensory Neurons to Relieve Cold Allodynia

**DOI:** 10.1523/ENEURO.0395-22.2022

**Published:** 2023-02-14

**Authors:** Federico Iseppon, Ana P. Luiz, John E. Linley, John N. Wood

**Affiliations:** 1 Molecular Nociception Group, Wolfson Institute for Biomedical Research, University College London, London WC1E 6BT, United Kingdom; 2 Discovery UK, Neuroscience, Biopharmaceuticals R&D, AstraZeneca, Cambridge CB21 6GH, United Kingdom

**Keywords:** oxaliplatin, pain, pregabalin, sensory neurons, silent nociceptors

## Abstract

Oxaliplatin is a platinum-based chemotherapeutic agent that causes cold and mechanical allodynia in up to 90% of patients. Silent Nav1.8-positive nociceptive cold sensors have been shown to be unmasked by oxaliplatin, and this event has been causally linked to the development of cold allodynia. We examined the effects of pregabalin on oxaliplatin-evoked unmasking of cold sensitive neurons using mice expressing GCaMP-3 in all sensory neurons. Intravenous injection of pregabalin significantly ameliorates cold allodynia, while decreasing the number of cold sensitive neurons by altering their excitability and temperature thresholds. The silenced neurons are predominantly medium/large mechano-cold sensitive neurons, corresponding to the “silent” cold sensors activated during neuropathy. Deletion of α2δ1 subunits abolished the effects of pregabalin on both cold allodynia and the silencing of sensory neurons. Thus, these results define a novel, peripheral inhibitory effect of pregabalin on the excitability of “silent” cold-sensing neurons in a model of oxaliplatin-dependent cold allodynia.

## Significance Statement

Pregabalin is an analgesic drug in the clinic, that is supposed to act by blocking neurotransmitter release. Here, we show that silent nociceptors that are activated by chemotherapeutic insults like oxaliplatin are silenced by pregabalin, which blocks the associated pain. This mode of action suggests that peripheral acting pregabalin-like drugs could be very useful for pain during chemotherapy, as they would have no CNS side effects, a problem for many patients with pregabalin. This novel effect of pregabalin is mediated by its interaction with the α2δ1 calcium channel subunit, but how this works is not yet understood.

## Introduction

Cold allodynia is a debilitating condition where patients experience mild, innocuous cooling as severe pain (Viana, 2018). This ailment is common in many neuropathic pain conditions, from nerve injury to diabetic neuropathy, toxin poisoning and chemotherapy treatment (MacDonald et al., 2020). Anticancer drugs, of which oxaliplatin is an example, are alkylating agents that act through the formation of DNA-platinum adducts to cause DNA damage (Cersosimo, 2005; Carozzi et al., 2015). Patients treated with platinum-based drugs usually develop acute allodynia as well as chronic neuropathic symptoms like numbness, ataxia, loss of reflexes, and of course pain: these side effects are often time and dose related, and in the case of oxaliplatin, they affect almost 90% of patients that undergo chemotherapy (Carozzi et al., 2015; MacDonald et al., 2020; Fumagalli et al., 2021).

The pathophysiological mechanisms of cold allodynia are currently unclear, but a recent article showed a common feature of many neuropathies carrying this condition: a previously unidentified population of large-diameter, silent cold-sensing neurons that become active and contribute to cold allodynia in these states (MacDonald et al., 2021). This shift in neuronal activity was particularly evident in an intraplantar pain model of oxaliplatin-induced neuropathy, one of the conditions that has the highest occurrence of peripheral cold allodynia (Deuis et al., 2013; Sałat et al., 2019; MacDonald et al., 2020, 2021). Oxaliplatin is a chemotherapeutic agent associated with acute neurotoxicity that manifests as mechanical and cold allodynia (Y. Yang et al., 2021). Oxaliplatin-dependent neurotoxicity may develop in two ways: an acute and reversible sensory neurotoxicity with cold-triggered paresthesia and dysesthesia of the extremities that occurs within hours from the infusion, and secondly, a chronic sensory neuropathy, again with a stocking-glove distribution, that occurs after accumulation of the drug because of repeated infusions (Cersosimo, 2005; Land et al., 2007; Carozzi et al., 2015).

There is still no clear therapeutic route to alleviate neuropathic pain symptoms and cold allodynia. Few drugs have shown promising results, but gabapentinoids show a promising analgesic function in several neuropathic pain models (Curros-Criado and Herrero, 2007; Han et al., 2007; Gustafsson and Sandin, 2009; Coderre et al., 2010; Mangaiarkkarasi et al., 2015; Chincholkar, 2018). These compounds are thought to act primarily on excitability through their interaction with voltage-gated calcium channels (VGCCs) via the auxiliary subunit α2δ1 (Bauer et al., 2009; Chincholkar, 2018), although they can have other molecular targets such as NMDA receptors (Verma et al., 2014; Carozzi et al., 2015; Alles and Smith, 2017; Chincholkar, 2018). Gabapentin, and its successor pregabalin, are thought to act both at spinal and supra-spinal loci, inhibiting ascending nociceptive input from dorsal root ganglion (DRG) neurons and activating descending inhibitor pathways to ameliorate neuropathic pain (Carozzi et al., 2015; Chincholkar, 2018). Its use, alone or in combination with other drugs, has been shown to be effective in numerous preclinical models of neuropathic pain (Verma et al., 2014). This drug has been shown to be particularly effective in the mitigation of cold allodynia, with single dose reversal of cold symptoms occurring after spinal nerve ligation (SNL) and spared-nerve injury (SNI) models as well as an attenuation of allodynia induced by oxaliplatin that showed pregabalin as the most efficacious drug alongside lidocaine and morphine as notable examples (Ling et al., 2008; Gustafsson and Sandin, 2009; Peng et al., 2012; Verma et al., 2014).

To investigate the functional effects of pregabalin on an acute, oxaliplatin-dependent model of cold allodynia, we used behavioral testing as well as *in vivo* calcium imaging to dissect the effect of this compound on DRG neuronal activity. Here, we describe a novel, peripheral action of pregabalin that results in the preferential silencing of the large-diameter, silent cold-sensing neurons involved in cold allodynia. Furthermore, we show that this effect is dependent on the α2δ1 subunit of the VGCCs and is abolished in a global α2δ1 transgenic knock-out (KO) mouse line.

## Materials and Methods

### Experimental model and treatment

#### Mice

All animal procedures conducted at University College London were approved by University College London ethical review committees and conformed to United Kingdom Home Office regulations (Animals (Scientific Procedures) Act 1986) under Project license P413329A2. For experiments using only wild-type (WT) mice, adult (more than six weeks old) C57BL/6 mice from Charles River were used. Both transgenic mice used in this paper had a C57BL/6 background. *Pirt*-GCaMP3 mice were kindly provided by Prof. Xinzhong Dong (John Hopkins University, Baltimore, MD; Kim et al., 2014) and global α2δ1 knock-out (KO) mice were generously donated by Prof. Annette Dolphin (University College London, London, United Kingdom; Patel et al., 2013).

For experiments using genetically altered lines, all were performed on adult male and female α2δ1-KO mice and wild-type littermates, obtained by breeding heterozygotes. Mice were housed in groups of no more than five on a 12/12 h light/dark cycle; food and water were available *ad libitum*. Both male and female animals were used for all experiments, in equal numbers where possible. These studies were not however designed to test for sex differences, and sexes were pooled for analysis. The number of animals used to generate each dataset is described in individual figure legends. For genotyping, genomic DNA was isolated from ear tissue followed by PCR. For *Pirt*-GCaMP3, the primers used were the following: forward: TCCCCTCTACTGAGAGCCAG; WT reverse: GGCCCTATCATCCTGAGCAC; GCaMP3 reverse: ATAGCTCTGACTGCGTGACC. For α2δ1-KO, the primers used were the following: primer 01: CATGGGTGGACAAGAT GCAGG; primer 02: CTGCACGAGACTA-GTGAGACG; primer 03: CATTCTCAAGACTGTA GGCAGAG.

### GCaMP virus injection

Pups from α2δ1 breeding heterozygote mice [postnatal day (P)2 to P5)] were used for injection of GCaMP-expressing virus. Before the injection, the mother was anesthetized with a low concentration of isoflurane (1%) and pups were anesthetized by hypothermia. Virus pAAV.CAG.GCaMP6s.WPRE.SV40 (plasmid #100844, Addgene) was injected into the plantar area of the hind paw using a 10-μl Hamilton syringe with a cannula connected to a 30-G needle; 5 μl of virus (titer = 5 × 10^12^ GC/ml) was injected into each of the two hindpaws. After injection, the pups were kept on a heating box until their body temperature returned to normal. Then they were put back into the cage before the mother was put back and rubbed against the pups. The mice injected with virus, after weaning, had the genotyping for α2δ1 subunit checked by PCR. Both sexes were used for *in vivo* imaging six to eight weeks after injection.

### Chemotherapy-induced neuropathy pain model

Chemotherapy-induced neuropathy was studied in mice using the intraplantar oxaliplatin model first described by Deuis and colleagues, because this treatment recapitulates the rapid onset of cold allodynia in human patients infused with the drug (Deuis et al., 2013). Oxaliplatin (Sigma, O9512) was dissolved in 5% glucose dH_2_O solution to an equivalent dose of 80 μg in 40 μl since oxaliplatin is unstable in chloride-containing saline solution. Mice were treated by intraplantar injection into the left hindpaw. Behavioral testing or imaging was assessed at least 3 h after injection.

### Treatment

After injection of oxaliplatin and the behavioral or calcium imaging baseline measurements (at least 3 h after injection), the mice were treated with either pregabalin (Sigma, Y0001805) at a dose of 2 mg/kg or vehicle (PBS) solutions via intravenous injection. Behavioral testing or calcium imaging were performed up to either 60 or 50 min after injection, respectively.

### Behavioral testing

All animal experiments were performed in accordance with Home Office Regulations. The investigator was blind to treatment and/or genotype. Animals were acclimatized to handling and every effort was made to minimize stress during the testing. Both male and female animals were used.

### Cold plate

Mice were placed on the Cold Plate apparatus (Ugo Basile). The Cold Plate was maintained at 10°C for 5 min while the animal was free to move around on the plate and the number of nociceptive behaviors (shaking, lifting, licking, guarding, biting) displayed by the injected hindpaw were counted by the observer (Luiz et al., 2019). In the experiments with animals that did not receive any intraplantar treatment, as the nociceptive behaviors are almost absent in the hindpaws, the number of forepaw lifts was assessed by the observer instead.

### *In vivo* calcium imaging

#### Acquisition

Adult mice expressing GCaMP3 (6–12 weeks, male and female) were anesthetized using ketamine (KETAVET; Zoetis; 100 mg/kg), xylazine (Rompun; Bayer; 15 mg/kg), and acepromazine (Elanco; 2.5 mg/kg). Depth of anesthesia was confirmed by pedal reflex and breathing rate. Animals were maintained at a constant body temperature of 37°C using a heated mat (VetTech). Lateral laminectomy was performed at spinal level L3–5. In brief, the skin was incised longitudinally, and the paravertebral muscles were cut to expose the vertebral column. Transverse and superior articular processes of the vertebrae were removed using OmniDrill 35 (WPI) and microdissection scissors. To obtain a clear image of the sensory neuron cell bodies in the ipsilateral dorsal root ganglion (DRG), the dura mater and the arachnoid membranes were carefully opened using microdissection forceps. Artificial spinal fluid (values are in mm: 120 NaCl, 3 KCl, 1.1 CaCl_2_, 10 glucose, 0.6 NaH_2_PO_4_, 0.8 MgSO_4_, and 18 NaHCO_3_, pH 7.4 with NaOH) was constantly perfused over the exposed DRG during the procedure to maintain tissue integrity. The animal was mounted onto a custom-made clamp attached to the vertebral column, rostral to the laminectomy. The trunk of the animal was slightly elevated to minimize interference caused by respiration. The DRG was isolated by coating with silicone elastomer.

Images were acquired using a Leica SP8 confocal microscope. A 10× dry, 0.4-N.A. objective with 2.2 mm working distanced was used, with image magnification of 0.75–3× optical zoom. GCaMP3 was excited using a 488 nm laser line (3–10% laser power). GCaMP was detected using a hybrid detector (60% gain). 512 × 512-pixel images were captured at a frame rate of 1.55 Hz, bidirectional scan speed of 800 Hz, and pixel dwell time of 2.44 μs.

Noxious and innocuous stimuli were applied to the left hindpaw, ipsilateral to the exposed DRG. For thermal stimuli, the ventral side of the paw was immersed with ice-water (nominally 0°C), acetone (100%) or water heated to 55°C using a Pasteur pipette. For delivery of precise temperature stimuli, a Peltier-controlled thermode (Medoc) was used. For mechanical stimuli, a pinch with serrated forceps was used. An interval of at least 30 s separated each stimulus application.

#### Analysis

Image stacks were registered to a reference image, typically, the first frame in the series, using the FIJI plugin TurboReg (accurate rigid body transformation) to correct for XY drift. Stacks that showed excessive Z movement were excluded from analysis. Regions of interest (ROIs) were manually drawn around apparently responding cells using the polygon tool in FIJI. Mean pixel intensity over time for each ROI was extracted and analyzed. The time series of mean pixel intensity for each ROI was smoothened by a five time point rolling average to remove high-frequency noise. Next, we calculated the derivative of the mean pixel intensity. We calculated a mean baseline derivative for the 20 s preceding stimulus application. Neurons were classed as responders if, within 30 s of stimulus application, the maximum derivative was greater than the baseline derivative plus four standard deviations, that is, a Z-score of at least 4. We then calculated the ΔF/F_0_ value for each response to obtain a normalized measure of change in fluorescence. Neurons which showed a ΔF/F_0_ <0.25 were then discarded. Each trace was then manually screened as a further precaution against false positives. The remaining neurons that constituted the responding population were then used for statistical analysis.

### Quantification and statistical analysis

For *in vivo* imaging experiments, *n* refers to the number of cells responding to any stimulus. For all imaging and data, the number of animals used is indicated in the legend. For behavioral experiments, n refers to the number of animals. No power calculations were performed; however, sample sizes are similar to those used in the field.

Datasets are presented using appropriate summary statistics as indicated in the legend, typically accompanied by raw data points or a representation of the underlying distribution. Behavioral data were generally assumed to be normal, as is typical in the field, and error bars denote mean ± SEM. When considering *in vivo* imaging experiments, for responding cell population comparisons, percentages were calculated individually from each animal. For polymodality, threshold, and area analyses cells from all animals were pooled. Normality was not assumed when comparing cross-sectional areas or response magnitude of responding cells. Data are summarized using averages and SEMs, as well as cumulative or numerical distribution plots.

Tests of statistical comparison for each dataset are described in detail in figure legends. When comparing two treatment timepoints, paired *t* test was used. When comparing the distribution of cell cross-sectional areas for two groups, the Wilcoxon test was used. For more than two groups, one-way ANOVA or Kruskal–Wallis test was used with *post hoc* tests corrected for multiple comparisons. When comparing the effect of two factors on multiple groups, a repeated measures two-way ANOVA was used, with *post hoc* tests corrected for multiple comparisons. Curve fitting was performed using linear or nonlinear regression functions.

Statistical tests were all performed using GraphPad Prism 9. An α-value of *p < *0.05 for significance testing was used. All *p*-values resulting from planned hypothesis testing are reported.

### Data and materials availability

The data that support the findings of this study are openly available at: https://figshare.com/articles/dataset/Pregabalin_silences_oxaliplatin-activated_sensory_neurons_to_relieve_cold_allodynia/20200109.

## Results

### Pregabalin ameliorates oxaliplatin-dependent cold allodynia by suppressing silent cold-sensing neurons

#### Pregabalin alleviates cold allodynia in oxaliplatin-dependent neuropathy

**Figure 1.**
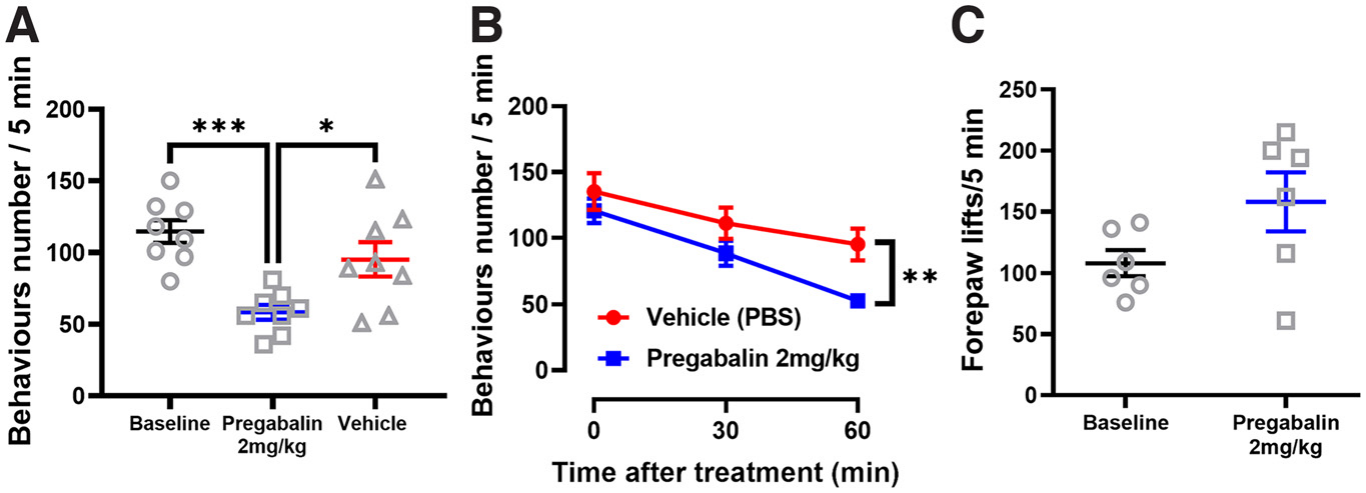
Assessment of the effect of pregabalin on acute oxaliplatin-induced cold allodynia. ***A***, Cold-plate assessment at 10°C of mice treated with oxaliplatin and successively either vehicle or 2 mg/kg of pregabalin. Activity was measured as the total number of nociceptive behaviors (hindpaw lifts, shakes, licks) over the test duration. A cutoff time of 300 s was used to limit tissue damage. The baseline data in the graph refers to the mice injected with oxaliplatin. For the baseline versus oxaliplatin-treated animal data please refer to Extended Data Figure 1-1. ***B***, Plot showing the decrease of the number of nociceptive behaviors over time following vehicle or pregabalin injection. ***C***, Cold-plate assessment at 10°C of mice treated only with pregabalin. Activity was measured as the total number of forepaw lifts over the test duration. A cutoff time of 300 s was used to limit tissue damage. *n *=* *8 oxaliplatin + pregabalin mice, *n *=* *8 oxaliplatin + vehicle mice, *n *=* *6 pregabalin only mice. Statistical analyses in ***A*** and ***B*** were performed using one-way and two-way ANOVA tests with multiple comparisons, respectively. Statistical Analysis in ***C*** was performed using paired *t* test. *p < 0.05, **p < 0.01, ***p < 0.001.

Cold allodynia following oxaliplatin treatment is a side effect that occurs in humans within a few hours from the injections. The model used in this paper, developed to isolate the activity of oxaliplatin on peripheral sensory neurons, consists of an intraplantar injection of 80 μg (in 40 μl) of oxaliplatin that elicits strong nociceptive responses, that comprise licking, flinching, and shaking of the injected paw as soon as 3–4 h from the injection, assessed using a cold plate assay (Deuis et al., 2013; MacDonald et al., 2021). We observed the same increase in nociceptive responses on oxaliplatin injection when performing a cold plate assay over 5 min at 10°C (Extended Data Fig. 1-1). When the mice were treated with 2 mg/kg of pregabalin via intravenous injection, the number of nociceptive behaviors decreased significantly from 115 (±7.9) to 58 (±5.1). Control mice injected with vehicle showed a slight decrease in the total number of behaviors with high variability and no significant difference from the untreated ones (95 ± 11.9; Fig. 1*A*). Nociceptive responses to cold temperature were measured 30 and 60 min after intraplantar injection, and a significant difference between the mice treated with pregabalin and those treated with vehicle is evident only after 60 min (Fig. 1*B*). Next, we assessed the specificity of the effect of pregabalin on cold allodynia by treating mice with 2 mg/kg pregabalin without the prior oxaliplatin injection. In this case, no difference was observed in the behavior of the mice maintained 5 min on a 10°C cold plate (from 108 ± 10.6 to 158 ± 24.2; Fig. 1*C*).

10.1523/ENEURO.0395-22.2022.f1-1 Extended Data Figure 1-1 Assessment of cold allodynia after injection of oxaliplatin. Cold-plate assessment at 10°C of mice treated with oxaliplatin. Activity was measured as the total number of nociceptive behaviors (shaking, lifting, licking, guarding, biting) over the test duration. A cutoff time of 300 s was used to limit tissue damage. *n *=* *10 oxaliplatin-treated mice. Statistical analysis was performed using paired *t* test. ****p < 0.0001. Download Figure 1-1, TIF file.

#### Pregabalin silences cold-sensing cells by decreasing their excitability

**Figure 2.**
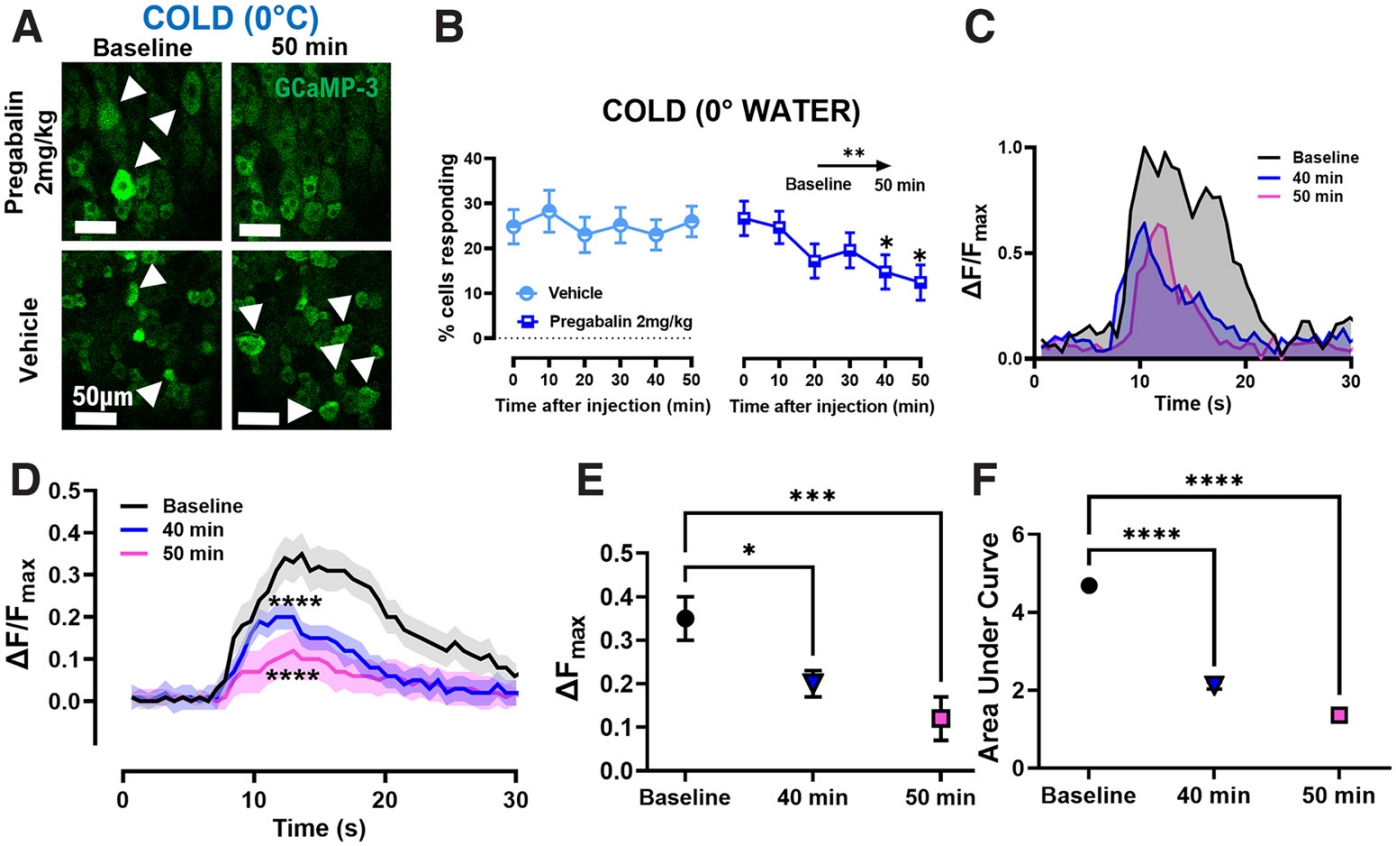
Pregabalin treatment decreases both the number of neurons responding to cold stimuli and the intensity of their responses. ***A***, Example images showing the reduction in the population of DRG neurons responding to ice-water 50 min after treatment with 2 mg/kg of pregabalin. ***B***, Graph showing the decrease of the percentages of cell responding to ice-water stimulus. ***C***, Example traces showing the decrease in the intensity of the calcium signals over time from pregabalin injection. ***D***, Plot showing the mean response amplitude before (black trace) and 40 min (blue) and 50 min (pink) after pregabalin injection. *n *= 45 cells for all time points. ***E***, Graphs showing the ΔF_max_ changes before and 40 and 50 min after pregabalin injection. ***F***, Graphs showing the area under curve changes before and 40 and 50 min after pregabalin injection. *n *=* *6 pregabalin-treated mice, *n *=* *5 vehicle-treated mice. Statistical analysis in ***B*** was performed using repeated measures ANOVA test with multiple comparisons. Statistical analysis in ***D*** was performed using multiple Wilcoxon tests. Statistical analyses in ***E*** and ***F*** were performed using one-way ANOVA. *p < 0.05, **p < 0.01, ***p < 0.001, ****p < 0.0001. For the data regarding acetone, hot water, and mechanical stimulations please refer to Extended Data Figures 2-1 and 2-2.

Next, to dissect the functional effect of pregabalin on cold allodynia, we used *in vivo* calcium imaging to investigate whether and how pregabalin treatment affects the responses of sensory neurons to various mechanical and thermal stimuli, with particular attention to any changes in their responses to cold temperatures. It is known that the acute oxaliplatin-dependent neuropathy model used in this study causes a change in the peripheral representation of cold, with the appearance of a population of large, normally silent, cold responding cells (MacDonald et al., 2021). Using laser-scanning confocal imaging, we performed one-photon calcium imaging on the L4 DRG of oxaliplatin-treated mice to image the calcium signals of the neurons’ somata. Our experiments show a similar result, with a population of cells responding to ice-water and acetone stimuli of 27% and 17%, respectively (Fig. 2*A*,*B*; Extended Data Fig. 2-1). Interestingly, after intravenous injection of 2 mg/kg of pregabalin, we observed a decrease of the percentage of cells responding to either ice-water or acetone that becomes significant as early as 40 min after injection and decreases to 13% and 8% 50 min after injection (Fig. 2*B*; Extended Data Fig. 2-1*B*). Moreover, following pregabalin treatment, we observed a decrease in the intensity of the calcium signals evoked in cold-responding cells: both the peak fluorescence intensity (ΔF_max_) and the area under the curve decreased significantly after 40–50 min from the injection of pregabalin (Fig. 2*C–F*).

10.1523/ENEURO.0395-22.2022.f2-1 Extended Data Figure 2-1 Pregabalin treatment decreases the number of neurons responding to a chemical cold stimulus. ***A***, Example images showing the reduction in the population of DRG neurons responding to acetone 50 min after treatment with 2 mg/kg of pregabalin. ***B***, Graph showing the decrease of the percentages of cell responding to acetone. *n *=* *6 pregabalin-treated mice, *n *=* *5 vehicle-treated mice. Statistical analysis in ***B*** was performed using repeated measures ANOVA test with multiple comparisons. *p < 0.05, **p < 0.01. Download Figure 2-1, TIF file.

10.1523/ENEURO.0395-22.2022.f2-2 Extended Data Figure 2-2 Pregabalin treatment does not affect other sensory modalities besides cold. ***A***, Example images showing no change in the population of DRG neurons responding to mechanical pinch 50 min after treatment with 2 mg/kg of pregabalin. ***B***, Graph showing the unchanged percentages of cell responding to mechanical pinch. ***C***, Example images showing no change in the population of DRG neurons responding to a 55°C water stimulus 50 min after treatment with 2 mg/kg of pregabalin. ***D***, Graph showing the unchanged percentages of cell responding to a 55°C water stimulus. *n *=* *6 pregabalin-treated mice, *n *=* *5 vehicle-treated mice. Statistical analysis in ***B*** and ***D*** was performed using repeated measures ANOVA test with multiple comparisons. Download Figure 2-2, TIF file.

This effect of pregabalin is specific to cold sensory stimuli, the ones most significantly impacted by oxaliplatin: we observed no significant change in the number of cells responding to either mechanical or heat stimulation at any point during the treatment with pregabalin (Extended Data Fig. 2-2).

**Figure 3.**
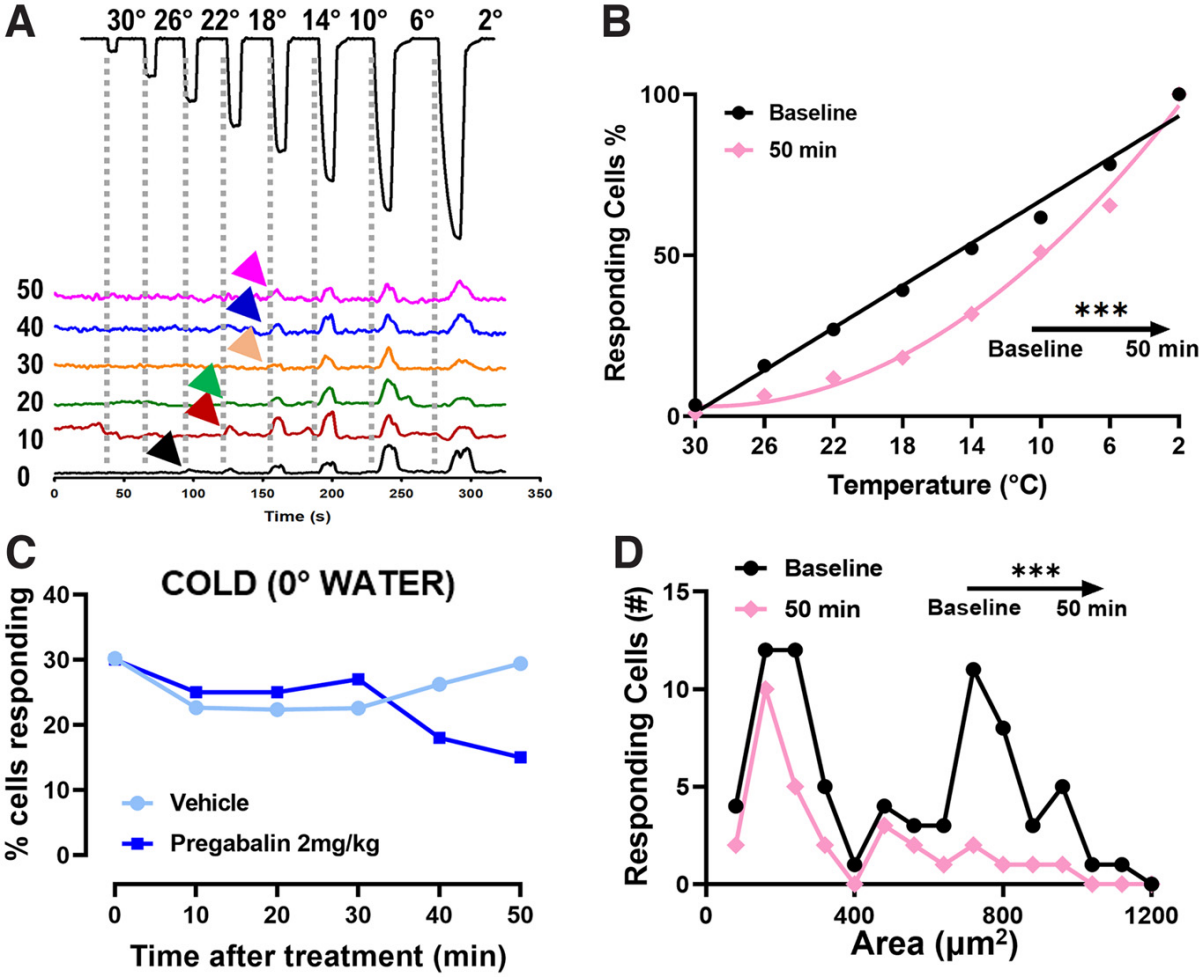
Pregabalin treatment significantly reduces the excitability of cold responding cells and targets preferentially medium-large mechano-cold sensors. ***A***, Example traces of a representative cold-sensing cell in response to a series of 4°C temperature drops from a holding temperature of 32°C. The traces represent the change in the threshold of this neuron over time (0–50 min) from pregabalin injection. Arrowheads mark the first response to temperature drops in every timepoint. ***B***, Graph showing the change of the relationship between the number of cold-responding neurons and the temperature drop over time from pregabalin injection. While the relationship at baseline can be fit with a linear equation (y = −3.292× + 99.84; *r*^2^ = 0.9880; *n *=* *115), the one at 50-min fits a quadratic equation (y = 110.8 − 7.381× + 0.1265x^2^; *r*^2^ = 0.9927; *n *=* *109). The slopes at baseline and 50 min after treatment are significantly different (***p < 0.001), as analyzed by Wilcoxon test. For a complete visualization of the data regarding the cold threshold changes in all time points please refer to Extended Data Figure 3-1. ***C***, Graphs showing the change in the number of total mechano-responding neurons that respond also to ice-water stimulus. Mice treated with vehicle do not exhibit the decrease over time from treatment as the mice treated with pregabalin do. For the graphs showing the mechano-acetone and mechano-heat responding cells trends please refer to Extended Data Figure 3-2. ***D***, Numeric plots of the distribution of cold-responding cell areas in oxaliplatin-treated mice before (black trace) and after 50 min (pink trace) from pregabalin injection. The difference between the distributions at different timepoints after treatment is statistically significant (***p = 0.0001), as analyzed by Wilcoxon test. For a complete visualization of the numeric distributions of cold responding cells areas in all time points please refer to Extended Data Figure 3-3. *n = 7* mice for temperature threshold experiments; *n *=* *6 pregabalin-treated mice, *n *=* *5 vehicle-treated mice.

The oxaliplatin treatment is known not to affect the temperature threshold or excitability of cold sensing neurons, while unmasking silent cold nociceptors (MacDonald et al., 2021). We investigated any possible change in the thermal activation thresholds of cold responding cells after treatment with pregabalin by delivering transient 4°C temperature drops, from 30°C to 2°C, through a Peltier-controlled thermode (Fig. 3*A*). While at baseline the cell recruitment rises in a linear fashion with temperature decrease, after treatment with pregabalin the linear correlation skews toward lower temperatures, this is a significant change and an indicator that pregabalin increases the activation threshold of cold responding cells (Fig. 3*B*; Extended Data Fig. 3-1).

10.1523/ENEURO.0395-22.2022.f3-1 Extended Data Figure 3-1 Pregabalin treatment increases temperature threshold of cold-responding cells over time. Graph showing the change of the relationship between the number of cold-responding neurons and the temperature drop over time from pregabalin injection. Download Figure 3-1, TIF file.

10.1523/ENEURO.0395-22.2022.f3-2 Extended Data Figure 3-2 Pregabalin treatment reduces the number of polymodal mechano-cold but not mechano-heat responding cells. ***A***, Graph showing the change in the number of total mechano-responding neurons that respond also to acetone stimulus. Mice treated with vehicle do not exhibit the decrease over time from treatment as the mice treated with pregabalin do. ***B***, Graph showing the change in the number of total mechano-responding neurons that respond also to a 55°C water stimulus. Mice do not exhibit significant differences in the percentage of polymodal mechano-heat responding neurons over time from pregabalin treatment. *n *=* *6 pregabalin-treated mice, *n *=* *5 vehicle-treated mice. Download Figure 3-2, TIF file.

10.1523/ENEURO.0395-22.2022.f3-3 Extended Data Figure 3-3 Pregabalin preferentially inhibits the activity of “silent” cold-sensing neurons. ***A***, Cumulative plots of the cell areas in oxaliplatin-treated mice before and after pregabalin injection. The difference between the distributions at different timepoints after treatment is statistically significant (*p* < 0.0001), as analyzed by Kruskal–Wallis test. ***B***, Number plots of the distribution of cell areas of cold-responding cells in oxaliplatin-treated mice before and after pregabalin injection. The difference between the numeric distributions at different timepoints after treatment is statistically significant (*p* < 0.0001), as analyzed by Kruskal–Wallis test. ***C***, Graph showing the area under curve changes before and 50 min after pregabalin injection. The cell areas are divided into basal cold responding cells (A < 480 μm^2^) and silent cold sensors (A > 480 μm^2^). Albeit there is a difference in both populations after 50 min from treatment, the population of silent cold sensors seems to be silenced almost completely. These thresholds have been calculated previously (MacDonald et al., 2021). Download Figure 3-3, TIF file.

#### Pregabalin treatment suppresses predominantly “silent” cold sensors

Acute oxaliplatin injection indeed causes the recruitment of cells that do not respond to cold stimuli before treatment. It also increases receptor polymodality, with a marked increase in neurons responding to both mechanical and cold stimuli: the population of silent mechano-sensors primarily respond to high-threshold mechanical stimuli (MacDonald et al., 2021). We observed similar percentages in our model, and furthermore the injection of pregabalin decreased the population of cells responding to both mechanical and ice-water and acetone from 30% to 15% and from 20% to 6%, respectively (Fig. 3*C*; Extended Data Fig. 3-2*A*). This effect is specific to mechano-cold responding cells, as the percentage of mechano-heat polymodal neurons did not vary significantly after treatment in either condition (Extended Data Fig. 3-2*B*). When analyzing the area of cold-responding neurons we find the same two different populations found in MacDonald et al. (2021). Pregabalin treatment significantly affects the peripheral representation of cold allodynia: the analysis of the cross-sectional area of cold-responding cells shows a significant difference between the area of responding neurons after 50 min from pregabalin injection. Indeed, the treatment, although not completely, preferentially silences larger cold-responding cells, those “silent” cold sensors that are unmasked in the oxaliplatin-dependent neuropathy (Fig. 3*D*; Extended Data Fig. 3-3).

Taken together, these results highlight the presence of a new, peripheral effect of pregabalin that inhibits the activity of de-novo cold sensing neurons by decreasing their excitability and thus ameliorates cold allodynia in a model of acute oxaliplatin-dependent neuropathy.

### The peripheral effect of pregabalin is dependent on the voltage-gated calcium channel subunit α2δ1

**Figure 4.**
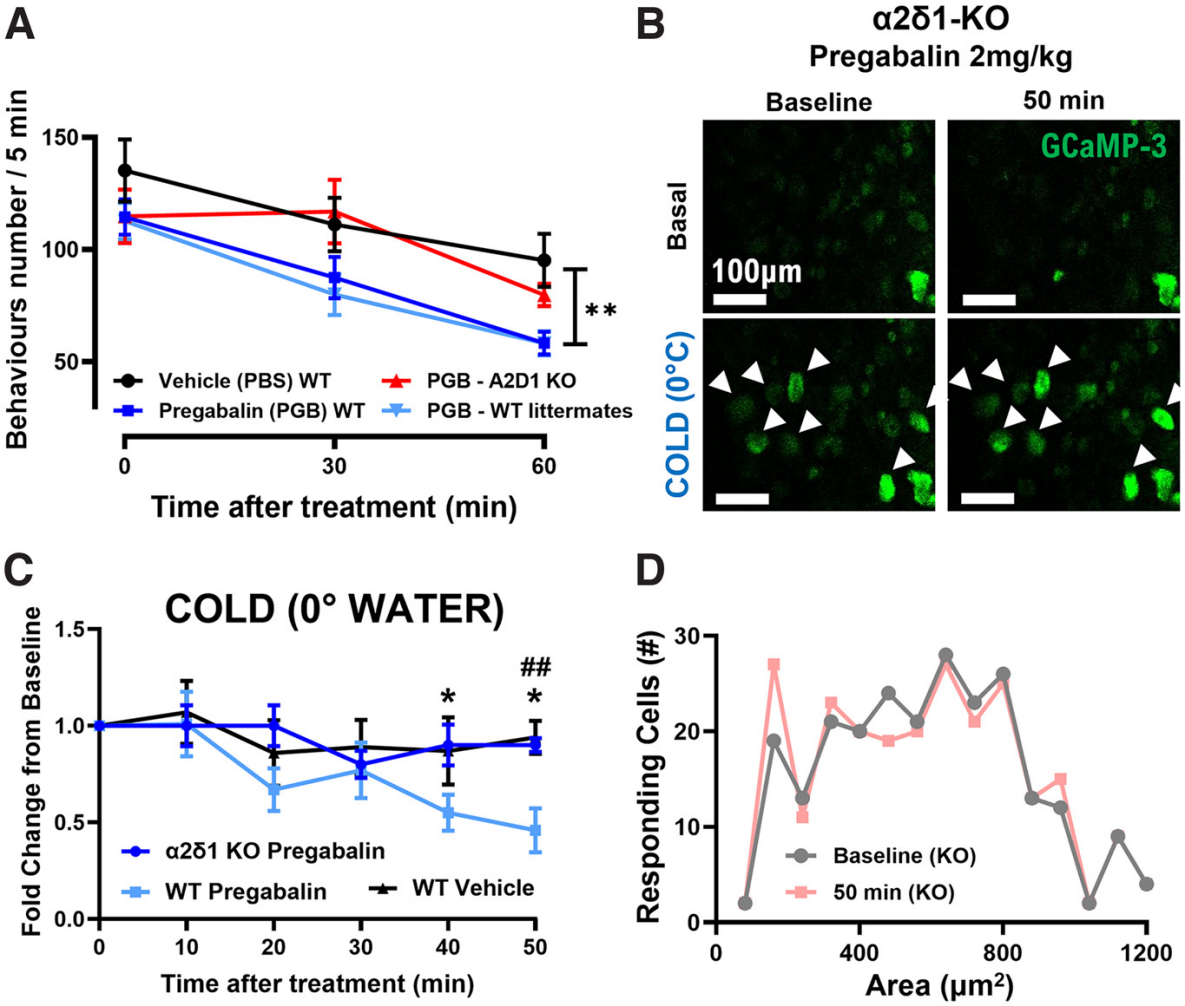
Knock-out of the α2δ1 subunit of the VGCCs is sufficient to abolish the effects of pregabalin both on cold allodynia and on silencing of cold responding DRG neurons. ***A***, Plot showing the decrease of the number of nociceptive behaviors over time from vehicle (*n *=* *8; black trace) or pregabalin injection in WT [*n *=* *8 (blue trace) + 10 (light blue trace)] and α2δ1-KO mice (*n *=* *10; red trace) when exposed to a 10°C cold plate for 5 min (**p < 0.01). ***B***, Example images showing that the reduction of the population of DRG neurons responding to ice-water 50 min after treatment with pregabalin is absent in α2δ1-KO mice. ***C***, Graph showing the disappearance of the decrease of the percentages of cell responding to ice-water stimulus in α2δ1-KO mice. Change is quantified as fold-change to baseline. ***D***, Numeric plots of the distribution of cold responding cell areas in oxaliplatin-treated α2δ1-KO mice before (gray trace) and 50 min after pregabalin injection (light pink trace). *n *=* *7 α2δ1-KO mice, *n *=* *6 pregabalin-treated mice, *n *=* *5 vehicle-treated mice for imaging data. Statistical analyses in ***C*** were performed using repeated measures ANOVA test with multiple comparisons. *p < 0.05 (WT vehicle vs WT pregabalin treated); ##*p* < 0.01 (α2δ1-KO vs WT pregabalin treated). For the data regarding acetone, hot water, and mechanical responses, please refer to Extended Data Figure 4-1. For the data regarding the area distribution of cold responding cells after pregabalin treatment in α2δ1-KO animals, please refer to Extended Data Figure 4-2.

Next, we investigated the mechanism of action of pregabalin on the silencing of the unmasked cold sensors by studying the role of its best-known target: α2δ1. We performed the same cold plate assay described before on WT and global α2δ1-KO mice: we treated all mice with oxaliplatin and 2 mg/kg of pregabalin, and we observed a significant difference between the two groups. Moreover, when the results were compared with the previous ones, there is an overlap between the vehicle-treated group and the α2δ1-KO mice, suggesting an almost complete loss of the effect of pregabalin (Fig. 4*A*).

10.1523/ENEURO.0395-22.2022.f4-1 Extended Data Figure 4-1 Knock-out of the α2δ1 subunit of the VGCCs abolishes the effects of pregabalin on cold but has no effect on heat and mechanical responses. ***A***, Example images showing no change in the population of DRG neurons responding to acetone, 55°C water, and mechanical pinch 50 min after treatment of α2δ1-KO mice with 2 mg/kg of pregabalin. ***B***, Graph showing the unchanged percentages of cell responding to acetone in α2δ1-KO mice with respect to WT ones. ***C***, Graph showing the unchanged percentages of cell responding to a 55°C water stimulus in α2δ1-KO mice with respect to WT ones. ***D***, Graph showing the unchanged percentages of cell responding to mechanical pinch in α2δ1-KO mice with respect to WT ones. *n *=* *7 α2δ1-KO mice, *n *=* *6 pregabalin-treated mice, *n *=* *5 vehicle-treated mice for imaging data. Statistical analyses in ***B–D*** were performed using repeated measures ANOVA test with multiple comparisons. ##*p* < 0.01 (α2δ1-KO vs WT pregabalin treated). Download Figure 4-1, TIF file.

10.1523/ENEURO.0395-22.2022.f4-2 Extended Data Figure 4-2 Knock-out of the α2δ1 subunit of the VGCCs abolishes the silencing effect of pregabalin on silent cold sensors. Graph showing the area under curve changes before and 50 min after pregabalin injection in the α2δ1-KO mice. The cell areas are divided into basal cold responding cells (A < 480 μm^2^) and silent cold sensors (A > 480 μm^2^). There seems to be no difference in the population of basal and silent cold sensors after pregabalin treatment when the α2δ1 subunit is knocked down globally. These thresholds have been calculated previously (MacDonald et al., 2021). Download Figure 4-2, TIF file.

We then proceeded with *in vivo* imaging experiments on α2δ1-KO mice. Similar to the results of the behavioral tests, the decrease of the percentage of cold-sensing neurons is completely abolished in the α2δ1-KO mice, both in the case of ice-water and acetone stimuli (Fig. 4*B*; Extended Data Fig. 4-1*A*,*B*). This effect of α2δ1 is specific again to cold stimuli, as the percentages of heat and mechanical-responding neurons did not vary significantly between conditions (Extended Data Fig. 4-1*A*,*C*,*D*). Furthermore, the cross-sectional area of the cold-responding neurons is similar to that of WT animals and did not vary on treatment with pregabalin (Fig. 4*D*; Extended Data Fig. 4-2), consistent with the behavioral results and suggesting a strong dependence of the effect of pregabalin on its main molecular target α2δ1.

## Discussion

### Pregabalin exerts a novel, peripheral effect on cold allodynia induced by oxaliplatin in mice

We have identified a novel effect of pregabalin on “silent” cold sensing neurons that acquire *de novo* sensitivity to cold after cold allodynia induction by acute injection of oxaliplatin. Pregabalin, in this model, acts peripherally on this subpopulation of peptidergic A-fiber nociceptors by decreasing their excitability and thus mitigating the increased responses to cold stimuli that is thought to be a common pathophysiology underlying cold allodynia in various models of neuropathic pain (MacDonald et al., 2021).

### Oxaliplatin mediates a *de novo* sensitization of previously silent nociceptors to cold stimuli: pregabalin reverses it

Chemotherapy-induced peripheral neuropathy (CIPN) is a side effect of oxaliplatin treatment, as it is for many anticancer therapies.

Many animal models of oxaliplatin-dependent neuropathy have been developed that replicate the acute and chronic symptoms experienced by human patients (Ling et al., 2008; Deuis et al., 2013; Yamamoto et al., 2016). We chose a model of acute neuropathy to focus the attention on the early changes in cold perception and understand the molecular basis of cold allodynia. Indeed, there is strong evidence of changes in the coding of the responses to cold stimuli in the DRG: in the healthy state, only a limited population of small-diameter neurons responds to a cooling stimulus in a very specific fashion. After treatment with oxaliplatin, cold stimuli elicit the response of a population of previously silent neurons responding to both cold and noxious mechanical stimuli. The investigation of their molecular identity suggests they could be classified as A-fiber peptidergic nociceptors that express the sodium channel NaV1.8, whose key role in the sensation of prolonged, extreme cold in normal conditions has been previously shown (Luiz et al., 2019; MacDonald et al., 2021).

Pregabalin is a well-known anti-epileptic and analgesic drug that has been one of the staples in the treatment of neuropathic pain for years. Pregabalin was effective in ameliorating CIPN in numerous clinical trials of cancer-related neuropathic pain and allodynia after treatments with vincristine, paclitaxel, and drug cocktails comprising oxaliplatin (Mishra et al., 2012; Atreya, 2016; Avan et al., 2018; Sugimoto et al., 2021). However, data regarding its effectiveness in the treatment of cold allodynia are more controversial: earlier clinical trials and case studies found clear effectiveness of pregabalin to treat oxaliplatin-dependent neuropathy, whereas a more recent double-blinded trial found no significant difference from placebo on oxaliplatin-dependent CIPN, and ASCO guidelines do not fully recommend the use of gabapentinoids to treat CIPN (Saif et al., 2010; de Andrade et al., 2017; Loprinzi et al., 2020).

Nevertheless, this article confirms previous data showing the effectiveness of pregabalin in treating oxaliplatin-dependent symptoms and highlights its potential role as a functional counter to the increased excitability caused by the chemotherapeutic treatment (Ling et al., 2008; Deuis et al., 2013; Starobova and Vetter, 2017). The injection of 2 mg/kg of pregabalin ameliorated cold allodynia symptoms with a significant decrease in the number of nocifensive responses of mice exposed to a 10°C cold plate assay. Furthermore, the treatment caused a significant decrease of the number of neurons responding to different cold stimuli within the DRG to almost normal levels, with a time frame that corresponds to the effect seen on the behavioral tests and is in concert with other experiments done in similar conditions (Ling et al., 2008; Deuis et al., 2014).

Furthermore, there seems to be a direct effect on cellular excitability: the temperature threshold of cells responding to cold, that is not affected by oxaliplatin, shifts significantly toward lower temperatures after treatment with pregabalin, suggesting that pregabalin may silence cold-sensing neurons by increasing their response threshold.

Finally, the investigation on the changes in polymodality and cross-sectional area of neurons responding to cold before and after pregabalin injection strongly indicates that, albeit not being a direct counter to the alterations elicited by oxaliplatin, pregabalin indeed inhibits preferentially the mechano-cold “silent” sensors that are the functional result of its pathologic action.

Taken together, these data suggest that pregabalin acts directly on DRG cells by reverting the functional representation of cold stimuli back to almost normal levels, and this in turn ameliorates the symptoms of cold allodynia.

### Pregabalin efficacy is dependent on the α2δ1 subunit of calcium channels

The main analgesic effect of pregabalin is assumed to be because of its action on the VGCCs through its direct binding with the α2δ1 subunit. This subunit is expressed in primary afferent fibers and is fundamental for a correct release of neurotransmitters and neuropeptides at the presynaptic site in the dorsal horn (DH) of the spinal cord (Hoppa et al., 2012; Dolphin, 2016; Alles and Smith, 2017). This protein has been extensively studied for its impact in neuropathic pain; it has been demonstrated to be upregulated in various models of neuropathic pain, and its upregulation alone is sufficient to drive allodynia, even in absence of a neuropathic injury (Li et al., 2006; Alles and Smith, 2017; Chincholkar, 2018). Furthermore, it was observed that knock-down of the α2δ1 subunit has an impact on acute sensation: α2δ1-KO mice in fact have impaired mechanical and cold sensitivity, as well as showing a marked delay in the onset of mechanical hypersensitivity in a neuropathic model of partial sciatic nerve ligation (PSNL). Moreover, in the same model, the absence of α2δ1 abolishes the anti-allodynic effect of a normally effective dose of pregabalin (Patel et al., 2013).

We do not observe any effect of the α2δ1 KO on the basal cold responses after oxaliplatin treatment, but we do show that the rapid effect of pregabalin on both the behavioral and functional representation of cold allodynia that were observed are almost completely abolished in the α2δ1-KO mice. Indeed, the same treatment that elicited a significant reduction both in the number of nocifensive behaviors and of cold-responding cells did not have any effect in the α2δ1-KO mice, further confirming the dependence of pregabalin on the α2δ1 molecule for its early action in this CIPN pain model.

### Site of action of pregabalin: central, peripheral, or both?

Although gabapentinoids have been widely used in curing patients with neuropathic symptoms, their mechanism of action is not fully understood. They are thought to act mainly by interfering with trafficking of CaV2.2 VGCCs to the presynaptic site of the dorsal laminae in the spinal cord, as well as blocking their function through the α2δ1 subunit, decreasing in turn the release of neurotransmitters and neuropeptides from DRG presynaptic sites and overall neuronal excitability in the DH (Verma et al., 2014; Chincholkar, 2018). However, decreased expression of VGCCs in the terminal may not be important, as their blockade by Mn^2+^ has a negligible effect on neurotransmitter release (Biggs et al., 2014; Alles and Smith, 2017). Other putative mechanisms of action include an inhibition of descending serotoninergic facilitatory and an excitation of descending noradrenergic inhibitory pathways, as well as an effect on synaptogenesis by blocking the interactions of thrombospondin to α2δ1 (Suzuki et al., 2005; Eroglu et al., 2009; Suto et al., 2014). Another recently discovered effect of gabapentinoids is the decrease of trafficking of NMDA glutamatergic receptors to presynaptic sites in the DH through a direct interaction of the C terminus of α2δ1 with these receptors (Chen et al., 2018). Other recently discovered binding partners of the α2δ1 subunits are neurexin-1α, whose weak interaction with α2δ1 increases presynaptic release, and calcium-activated potassium (BK) channels, which competes with α2δ1 for the binding to calcium channels and reduces overall calcium currents (Zhang et al., 2018; Alles et al., 2020; Taylor and Harris, 2020). These recent finds have highlighted how the signaling cascade downstream α2δ1, and subsequently the range of effects of pregabalin, may be much more complex than previously thought. Indeed, taken together, these findings show how pregabalin, thanks to its interaction with the α2δ1 subunit, has many different targets and sites of action throughout the nervous system, corroborated also by the fact that an increase of α2δ1 expression has been observed in multiple sites within the peripheral and central nervous system (Luo et al., 2001; Cole et al., 2005; Bauer et al., 2009; Yamamoto et al., 2016).

The principal site of action of pregabalin has been thought to be central, mainly at the spinal cord level but without ruling out the possibility of supra-spinal actions (Bauer et al., 2009; Verma et al., 2014; Chincholkar, 2018). Indeed, electrophysiological studies on intact and spinalized rat showed that the inhibitory effect observed with pregabalin treatment on C-fiber mediated nociceptive activity is abolished in spinalized animals, suggesting a strong involvement of supra-spinal centres (You et al., 2009). Nevertheless, our data strongly suggest a direct effect on the excitability of a specific subpopulation of peripheral DRG neurons that acquire cold sensitivity after oxaliplatin-dependent neuropathic injury. Albeit we cannot infer a direct causal effect between the antiallodynic effect of pregabalin and the decrease in the population of cold-sensing cells at the DRG level, we suggest a strong correlation between the behavioral data and the functional changes in the peripheral representation of noxious cold stimuli. Indeed, it has been observed that gabapentinoids have a direct effect on the activity of specific DRG neuronal populations: gabapentin inhibits persistent sodium currents in medium sized neurons, and has an inhibitory effect exerted preferentially on small IB4-negative and medium sized neurons (R.H. Yang et al., 2009; Biggs et al., 2014). The subpopulation of neurons preferentially hit by pregabalin, as shown in this paper, seems to be the silent cold sensors described previously (MacDonald et al., 2021); they are medium sized neurons, positive for Nav1.8 and CGRP. These results are further supported by microarray data on DRG of Nav1.8-DTA mice that show a strong reduction in the transcripts for CGRP-α and CGRP-β, as well as in CaV2.2 and α2δ1 (Abrahamsen et al., 2008). Furthermore, it has been shown that gabapentinoids influence neurons of the substantia gelatinosa in the DH, with an overall decrease in excitability that cannot be explained just by a decrease of calcium currents, since, as was said previously, their blockade by Mn^2+^ does not affect overall neurotransmitter release in the DH (Biggs et al., 2014). On the other hand, IB4-negative neurons and Aδ fibers from medium-sized neurons project primarily to excitatory DH neurons (Lu and Perl, 2005; Braz et al., 2014); these cells may be preferentially targeted by gabapentinoids, but we have also shown that the central action of pregabalin may in part depend on its selective inhibition of a specific subpopulation of neurons in the DRG.

These findings thus support a peripheral locus of action of gabapentin in diminishing the cold allodynia evoked by chemotherapeutic insults. The long-term nature of changes associated with neuronal damage and the clinical utility of peripheral application of gabapentin have still to be assessed. The precise mechanism of α2δ1-dependent analgesia evoked by gabapentinoids is also an important topic of future research. Nevertheless, the selective action of gabapentin on unmasked silent nociceptors highlights the role of silent nociceptors in cold allodynia and defines them as a potentially significant clinical target.

## Synthesis

Reviewing Editor: Christina Zelano, Northwestern University - Chicago Decisions are customarily a result of the Reviewing Editor and the peer reviewers coming together and discussing their recommendations until a consensus is reached. When revisions are invited, a fact-based synthesis statement explaining their decision and outlining what is needed to prepare a revision will be listed below. The following reviewer(s) agreed to reveal their identity: Uhtaek Oh.

The reviewers and I agree that this study is interesting and important. Although pregabalin is used widely for pain treatment, its mechanism of action is not fully understood. This manuscript provides insight into possible mechanisms of pregabalin’s mode of action on nociceptive sensory neurons. However, the reviewers raised important concerns that need to be addressed. Of particular importance are questions about the cold sensing neurons (see points 2 and 3 from Reviewer 1 below):

Reviewer 1:

This study focuses upon the effects of pregabalin, a widely used drug for pain which is also used as an anti-epileptic. It has been assumed that pregabalin works mainly in the CNS, but the findings here show that nociceptive neurons are one of the main targets. The novel points are the action of pregabalin in the peripheral nervous system with the finding that there are silent nociceptors during cold allodynia. The presence of silent cold receptors is an intriguing observation. The results are well presented and the subject of cold-sensing cells in the periphery is of interest. The dissection of the mechanism of action of pregabalin is a valuable approach. Several questions are raised by this investigation-

1) The oxaliplatin cancer agent is shown to produce an effective pain model of cold allodynia. Are there other agents that are associated with neuropathy from cold allodynia? This will solidify the chemotherapeutic effects and may reveal other mechanisms for the development of cold allodynia.

2) The cold sensing neurons are characterized by expression of Nav 1.8 and CGRP, but it would helpful to have additional markers for the sensory neurons. This is a concern with the findings that pregabalin reduces the population of cells responding to mechanical ice-water and acetone. What peripheral cells are more vulnerable? To distinguish these effects, more information is needed. Which populations are targets? Is there overlap in the populations as the data suggest. Do these cells express TRPM8 or ANKTM1 or TRPA1, which are reputed to display cold sensitivity?

3) The calcium channel α2δ1 subunit, sodium channels, NMDA channels and many other molecules are mentioned as potential downstream interactors. The VGCC is thought to possibly be a receptor for pregabalin. It is not certain how strong these interactions are for pregabalin. In addition, there are a number of holes in the signal transduction of pregabalin, which should be discussed.

4) The efficacy of pregabalin against pain in mice is discussed, but the exact doses and timing are not firmly established. As pregabalin has been used clinically for 20 years, it would be advantageous to use human data to help to assess the effectiveness of pregabalin.

Reviewer 2:

The authors describe the effect of pregabalin on oxaliplatin-induced neuropathic pain. The authors showed that pregabalin reduced oxaliplatin-evoked cold allodynia. They also showed with in vivo Ca2+ imaging that pregabalin reduced the number of DRG neurons responding to cold challenge. These behavioral and neuronal effects of pregabalin were not observed in α2δ1 KO mice. The paper contains important experimental results that may lead to a clue to treat chemotherapy induced neuropathic pain. But, the following points need to be answered.

1. The data in Fig. 1C look as if pregabalin causes nociception if the authors repeat the experiment. If so the author need to explain it.

2. Discussion is too long. The Discussion section contains unnecessary explanations. Some parts of the section belong to the Introduction such as actions or effects of CIPN, oxaliplatin, and pregabalins. It would be better if Discussion is short containing explanation of the meaning of the present data.

Minor points

1. Legends in Fig 1A. The baseline data in the graph refers to the mice injected with pregabaiin, which might be oxaliplatin?

2. Fig 1.3A, ’E’ was missing at ’xamples’, ∼∼

3. Page 5, line 8 from the bottom, ’dorsal horn (DH)’.

4. In Discussion page lines 5-6, “The principal site of action of pregabalin has been thought to be central, mainly at the spinal cord level but without ruling out the possibility of supra-spinal actions(12-14)”. This is a bit non-sense. The action of pregabalin is thought to be supraspinal, then why the authors rule out the supra-spinal actions?

## Author Response

Synthesis of Reviews:

Computational Neuroscience Model Code Accessibility Comments for Author (Required):

NA

Synthesis Statement for Author (Required):

The reviewers and I agree that this study is interesting and important. Although pregabalin is used widely for pain treatment, its mechanism of action is not fully understood. This manuscript provides insight into possible mechanisms of pregabalin’s mode of action on nociceptive sensory neurons. However, the reviewers raised important concerns that need to be addressed. Of particular importance are questions about the cold sensing neurons (see points 2 and 3 from Reviewer 1 below):

Reviewer 1:

This study focuses upon the effects of pregabalin, a widely used drug for pain which is also used as an anti-epileptic. It has been assumed that pregabalin works mainly in the CNS, but the findings here show that nociceptive neurons are one of the main targets.

The novel points are the action of pregabalin in the peripheral nervous system with the finding that there are silent nociceptors during cold allodynia. The presence of silent cold receptors is an intriguing observation. The results are well presented and the subject of cold-sensing cells in the periphery is of interest. The dissection of the mechanism of action of pregabalin is a valuable approach. Several questions are raised by this investigation 1) The oxaliplatin cancer agent is shown to produce an effective pain model of cold allodynia. Are there other agents that are associated with neuropathy from cold allodynia? This will solidify the chemotherapeutic effects and may reveal other mechanisms for the development of cold allodynia.

A) We thank the reviewer for this suggestion. Cold allodynia is a side effect that is common to many conditions and diseases that have neuropathic symptoms. Other chemotherapeutic agents like vincristine and cisplatin cause mainly acute mechanical allodynia, without cold symptoms, that seems to be peculiar to oxaliplatin treatment.

However, we previously showed that this mechanism of recruiting of silent sensors is common to other neuropathic pain conditions, caused by ciguatoxin or injury to the sciatic nerve (MacDonald et al., 2021). These neuropathic pain conditions all share the development of cold allodynia and the recruitment of silent cold sensing cells, albeit to different extent, nonetheless suggesting a common underlying pathophysiology.

2) The cold sensing neurons are characterized by expression of Nav 1.8 and CGRP, but it would helpful to have additional markers for the sensory neurons. This is a concern with the findings that pregabalin reduces the population of cells responding to mechanical ice water and acetone. What peripheral cells are more vulnerable? To distinguish these effects, more information is needed. Which populations are targets? Is there overlap in the populations as the data suggest. Do these cells express TRPM8 or ANKTM1 or TRPA1, which are reputed to display cold sensitivity?

A) We thank the reviewer for this insightful comment. Nav1.8-positive cells’ transcripts were analysed in our previous experiments and express little overlap with the TRPM8 channel, as TRMP8 is mainly expressed in cold sensing cells that activate in basal conditions and have a small diameter, unlike the larger silent cold nociceptors. In fact, most cold sensing neurons responding to temperatures >1{°}C are indeed Nav1.8-negative(Luiz et al., 2019). In the same analysis, no selective enrichment or depletion of the TRPA1 (previously known as ANKTM1) channel has been observed in the subpopulation of Nav1.8-positive sensory neurons. There is considerable evidence of a role for the TRPA1 channel in oxaliplatin-dependent cold allodynia, but the oxaliplatin-dependent neuropathy model is slightly different from the one we used. In their paper, Zhao et al. found a clear effect of TRPA1-KO as well as the channel inhibitor HC-030031 in cold allodynia (delivered by a single intra-peritoneal injection) (Zhao et al., 2012). However, in the oxaliplatin-dependent cold allodynia model (delivered by a single intra-plantar injection) neither the channel blocker nor the knockout of TRPA1 have any effect in ameliorating cold allodynia (Deuis et al., 2013). These differences between models (mainly injection sites and doses) may account for the differences seen in their dependency on TRPA1. A much higher dose (5-10 mg/kg) was needed to elicit cold allodynia via intraperitoneal injection, whereas only 1.6-2 mg/kg was sufficient via intraplantar injection, a concentration that is much closer to the one used in humans (2.5-3.5 mg/kg). Nav1.9, another target that has been proven important for the development of cold allodynia induced by a single, intraperitoneal injection of oxaliplatin (6 mg/kg), seems not to be required for the induction of cold allodynia (Deuis et al., 2013; Lolignier et al., 2015).

The anti-allodynic effect of pregabalin may not be due to specific subpopulations expressing canonical cold sensing cell markers like TRPM8 (absent from the Nav1.8 positive neurons and present on the basal, small cold sensors) or TRPA1, but maybe more related to the physiological causes of the activation of these silent sensors. Changes in potassium channel activity may be important, and we speculate that pregabalin may act on these channels indirectly via α2δ1-dependent effects. Indeed, we previously found that AP-4, a potassium channels blocker, and κ-dendrotoxin, a blocker specific for Kv1.1 potassium channels, cause an increase in cold sensing cells similar to the one that occurs upon oxaliplatin treatment (MacDonald et al., 2021). Importantly, we found enrichment of the transcripts Kcnk1 and Kcnk2 (encoding potassium channels Kv1.1 and 1.2 respectively) in the Nav1.8-positive cell population (Luiz et al., 2019).

Our focus for the future is therefore to work on a better understanding of the precise mechanisms that underlie the peripheral effect of pregabalin, to find more precise molecular markers for the silent cold sensor population, and to understand whether peripheral-restricted drugs may retain more promising therapeutic effects than previously thought, alongside diminished side effects.

3) The calcium channel α2δ1 subunit, sodium channels, NMDA channels and many other molecules are mentioned as potential downstream interactors. The VGCC is thought to possibly be a receptor for pregabalin. It is not certain how strong these interactions are for pregabalin. In addition, there are a number of holes in the signal transduction of pregabalin, which should be discussed.

A) We thank the reviewer for this suggestion. Pregabalin, as gabapentin, were created as a potential GABA mimetic, albeit they show no binding to these receptors. However, deletion of the α2δ1 calcium channel subunit blocks the action of gabapentin (Patel et al., 2013). Although limited evidence has been shown of direct effects of pregabalin on NMDA receptors and sodium currents, is common belief that the main actuators of its effects are the α2δ subunits of calcium channels, in particular α2δ1 (Chincholkar, 2018; Dolphin, 2016). Most of the newly added complexity to the pregabalin signalling cascade is closely related to the expansion of the binding partners of the α2δ1subunit beyond VGCCs.

NMDA receptors, potassium BK channels, and many other targets like thrombospondins, and α-neurexins can be all indirect targets of pregabalin through their newly found interactions with the α2δ1 protein (Alles et al., 2020). In our work, we found a novel, peripheral site of action s of pregabalin, that is also mediated via α2δ1. We then proceed to locate a preferential target in the NaV1.8-positive silent cold nociceptors unmasked by the oxaliplatin-dependent cold symptoms. At this point, with our results we can only speculate on the precise signalling mechanism of pregabalin that causes this silencing, we can only state that it is dependent on its main target, α2δ1. These considerations have been summarised and added to the discussion section.

4) The efficacy of pregabalin against pain in mice is discussed, but the exact doses and timing are not firmly established. As pregabalin has been used clinically for 20 years, it would be advantageous to use human data to help to assess the effectiveness of pregabalin.

A) We thank the reviewer for this suggestion. Pregabalin has been indeed used clinically to treat neuropathic pain symptoms in humans in a wide range of conditions. In Chemotherapy-Induced Peripheral Neuropathy (CIPN), pregabalin has been shown to be active in numerous clinical trials, ameliorating mechanical hypersensitivity and pain scores in cancer-related neuropathic pain, as well as treatments with vincristine, paclitaxel, taxane, and anti-cancer cocktails that comprised oxaliplatin (Atreya, 2016; Avan et al., 2018; Mishra et al., 2012; Sugimoto et al., 2021). These successful trials relied on similar doses and timings, administering between 75 and 600 mg/day in consecutive increments over a period of 2-6 weeks. However, these clinical data refer more to general pain scores and mechanical allodynia, rather than cold allodynia. Data regarding its effectiveness in oxaliplatin-dependent pain are controversial: a recent double-blind trial did not show any significant effects of pregabalin pre-treatment on oxaliplatin-dependent CIPN (de Andrade et al., 2017). Moreover, the ASCO guidelines did not recommend gabapentinoids as a treatment for CIPN, albeit recently their decision has been changed in moderate recommendation but only if duloxetine treatment has failed (Loprinzi et al., 2020).

However, earlier clinical trials demonstrated the efficacy of pregabalin in the treatment of oxaliplatin-dependent CIPN (SAIF et al., 2010). Thus, although the efficacy of pregabalin in pre-clinical models is clear, the efficacy in treating human patients is still controversial. This may be due to differences in the measurement and scoring of pain, as demonstrated in this work, where they observed that general pain scores in human patients suffering from CIPN from oxaliplatin, docetaxel, and paclitaxel treatment, were not significantly different than the placebo-treated cohort, but measurements of specific symptoms like pain evoked by touch, pressing pain or tingling showed a significant improvement after pregabalin treatment (Hincker et al., 2019). These observations have been summarised and added to the discussion in a dedicated section.

Reviewer 2:

The authors describe the effect of pregabalin on oxaliplatin-induced neuropathic pain. The authors showed that pregabalin reduced oxaliplatin-evoked cold allodynia. They also showed with in vivo Ca2+ imaging that pregabalin reduced the number of DRG neurons responding to cold challenge. These behavioral and neuronal effects of pregabalin were not observed in α2δ1 KO mice. The paper contains important experimental results that may lead to a clue to treat chemotherapy induced neuropathic pain. But, the following points need to be answered.

1. The data in Fig. 1C look as if pregabalin causes nociception if the authors repeat the experiment. If so the author need to explain it.

A) We thank the reviewer for this comment. The data in figure 1C refers to the effect of pregabalin on cold sensation of naïve mice without any oxaliplatin treatment. As cold allodynia is dependent on oxaliplatin injection (Figures 1A and B), untreated mice have almost no nociceptive reaction in the hindpaws when placed for 5 minutes on a 10{°}C cold plate. Therefore, both groups depicted in figure 1C do not express any difference in the nociceptive reaction of the hindpaws. That’s why we resorted in the observation of the forepaw lifts, which we normally don’t use as they are more variable within each mouse, and in this case seem to depict a slight but not significant increase in the number of the mice treated with pregabalin. This figure is to highlight the specific effect of pregabalin as an anti-allodynic drug, that has no significant effect on normal mice.

2. Discussion is too long. The Discussion section contains unnecessary explanations. Some parts of the section belong to the Introduction such as actions or effects of CIPN, oxaliplatin, and pregabalins. It would be better if Discussion is short containing explanation of the meaning of the present data.

A) We thank the reviewer for these suggestions. We tried to expand the introduction and reduce the discussion sections, as suggested. However, the length of the discussion section is due to preliminary explanation about our choices of neuropathy model and drug that paint a context that we feel is clearer and would be lost if some of this information was moved to the introduction. These sections were our way of connecting these novel findings with our previous observations about silent cold sensors and the wider, complex literature about oxaliplatin-dependent neuropathy and treatment.

Minor points

1. Legends in Fig 1A. The baseline data in the graph refers to the mice injected with pregabaiin, which might be oxaliplatin?

2. Fig 1.3A, ’E’ was missing at ’xamples’, ∼∼

3. Page 5, line 8 from the bottom, ’dorsal horn (DH)’.

4. In Discussion page lines 5-6, “The principal site of action of pregabalin has been thought to be central, mainly at the spinal cord level but without ruling out the possibility of supra-spinal actions(12-14)”. This is a bit non-sense. The action of pregabalin is thought to be supraspinal, then why the authors rule out the supra-spinal actions?

A) We thank the reviewer for this comment. We amended all the minor points correctly pointed out by the reviewer. As for point (4), our aim in that section of the discussion is not to rule out any supra-spinal actions, but rather highlight how pregabalin, a drug that has always been deemed to exclusively target the central nervous system, has a clear peripheral effect on silent DRG neurons that become activated after the oxaliplatin treatment, and speculate about the fact that in some cases central effects may be the result of peripheral changes in the coding of somatosensation and pain often neglected.

References

Alles, S. R. A., Cain, S. M., & Snutch, T. P. (2020). Pregabalin as a Pain Therapeutic: Beyond Calcium Channels. Frontiers in Cellular Neuroscience, 14. https://doi.org/10.3389/FNCEL.2020.00083

Atreya, S. (2016). Pregabalin in Chemotherapy Induced Neuropathic Pain. Indian Journal of Palliative Care, 22(1), 101. https://doi.org/10.4103/0973-1075.173941

Avan, R., Janbabaei, G., Hendouei, N., Alipour, A., Borhani, S., Tabrizi, N., & Salehifar, E. (2018). The effect of pregabalin and duloxetine treatment on quality of life of breast cancer patients with taxane-induced sensory neuropathy: A randomized clinical trial. Journal of Research in Medical Sciences : The Official Journal of Isfahan University of Medical Sciences, 23(1). https://doi.org/10.4103/JRMS.JRMS_1068_17

Chincholkar, M. (2018). Analgesic mechanisms of gabapentinoids and effects in experimental pain models: a narrative review. https://doi.org/10.1016/j.bja.2018.02.066

de Andrade, D. C., Jacobsen Teixeira, M., Galhardoni, R., Ferreira, K. S. L., Braz Mileno, P., Scisci, N., Zandonai, A., Teixeira, W. G. J., Saragiotto, D. F., Silva, V., Raicher, I., Cury, R. G., Macarenco, R., Otto Heise, C., Wilson Iervolino Brotto, M., Andrade de Mello, A., Zini Megale, M., Henrique Curti Dourado, L., Mendes Bahia, L., ... de Souza, Â. M. (2017). Pregabalin for the Prevention of Oxaliplatin-Induced Painful Neuropathy: A Randomized, Double-Blind Trial. The Oncologist, 22(10), 1154-e105. https://doi.org/10.1634/THEONCOLOGIST.2017-0235

Deuis, J. R., Zimmermann, K., Romanovsky, A. A., Possani, L. D., Cabot, P. J., Lewis, R. J., & Vetter, I. (2013). An animal model of oxaliplatin-induced cold allodynia reveals a crucial role for Nav1.6 in peripheral pain pathways. Pain, 154(9), 1749-1757. https://doi.org/10.1016/J.PAIN.2013.05.032

Dolphin, A. C. (2016). Voltage-gated calcium channels and their auxiliary subunits: physiology and pathophysiology and pharmacology. The Journal of Physiology, 594(19), 5369-5390. https://doi.org/10.1113/JP272262

Hincker, A., Frey, K., Rao, L., Wagner-Johnston, N., Abdallah, A. ben, Tan, B., Amin, M., Wildes, T., Shah, R., Karlsson, P., Bakos, K., Kosicka, K., Kagan, L., & Haroutounian, S. (2019). Somatosensory predictors of response to pregabalin in painful chemotherapy-induced peripheral neuropathy: a randomized, placebo-controlled, crossover study. Pain, 160(8), 1835-1846. https://doi.org/10.1097/J.PAIN.0000000000001577

Lolignier, S., Bonnet, C., Gaudioso, C., Noël, J., Ruel, J., Amsalem, M., Ferrier, J., Rodat-Despoix, L., Bouvier, V., Aissouni, Y., Prival, L., Chapuy, E., Padilla, F., Eschalier, A., Delmas, P., & Busserolles, J. (2015). The Nav1.9 channel is a key determinant of cold pain sensation and cold allodynia. Cell Reports, 11(7), 1067-1078. https://doi.org/10.1016/J.CELREP.2015.04.027

Loprinzi, C. L., Lacchetti, C., Bleeker, J., Cavaletti, G., Chauhan, C., Hertz, D. L., Kelley, M. R., Lavino, A., Lustberg, M. B., Paice, J. A., Schneider, B. P., Lavoie Smith, E. M., Smith, M. lou, Smith, T. J., Wagner-Johnston, N., & Hershman, D. L. (2020). Prevention and management of chemotherapy-induced peripheral neuropathy in survivors of adult cancers: ASCO guideline update. Journal of Clinical Oncology, 38(28), 3325-3348. https://doi.org/10.1200/JCO.20.01399

Luiz, A. P., MacDonald, D. I., Santana-Varela, S., Millet, Q., Sikandar, S., Wood, J. N., & Emery, E. C. (2019). Cold sensing by Na V 1.8-positive and Na V 1.8-negative sensory neurons. Proceedings of the National Academy of Sciences of the United States of America, 116(9), 3811-3816. https://doi.org/10.1073/pnas.1814545116

MacDonald, D. I., Luiz, A. P., Iseppon, F., Millet, Q., Emery, E. C., & Wood, J. N. (2021). Silent cold-sensing neurons contribute to cold allodynia in neuropathic pain. Brain. https://doi.org/10.1093/brain/awab086

Mishra, S., Bhatnagar, S., Goyal, G. N., Rana, S. P. S., & Upadhya, S. P. (2012). A Comparative Efficacy of Amitriptyline, Gabapentin, and Pregabalin in Neuropathic Cancer Pain: A Prospective Randomized Double-Blind Placebo-Controlled Study. American Journal of Hospice and Palliative Medicine, 29(3), 177-182. https://doi.org/10.1177/1049909111412539/ASSET/IMAGES/LARGE/10.1177_1049909111412539

-FIG2.JPEG

Patel, R., Bauer, C. S., Nieto-Rostro, M., Margas, W., Ferron, L., Chaggar, K., Crews, K., Ramirez, J. D., Bennett, D. L. H., Schwartz, A., Dickenson, A. H., & Dolphin, A. C. (2013). α2δ-1 gene deletion affects somatosensory neuron function and delays mechanical hypersensitivity in response to peripheral nerve damage. The Journal of Neuroscience : The Official Journal of the Society for Neuroscience, 33(42), 16412-16426. https://doi.org/10.1523/JNEUROSCI.1026-13.2013

SAIF, M. W., SYRIGOS, K., KALEY, K., & ISUFI, I. (2010). Role of Pregabalin in Treatment of Oxaliplatin-induced Sensory Neuropathy. Anticancer Research, 30(7), 2927. http://ar.iiarjournals.org/content/30/7/2927.abstract

Sugimoto, M., Takagi, T., Suzuki, R., Konno, N., Asama, H., Sato, Y., Irie, H., Okubo, Y., Nakamura, J., Takasumi, M., Hashimoto, M., Kato, T., Kobashi, R., Hikichi, T., & Ohira, H. (2021). Mirogabalin vs pregabalin for chemotherapy-induced peripheral neuropathy in pancreatic cancer patients. BMC Cancer, 21(1). https://doi.org/10.1186/S12885-021-09069-9

Zhao, M., Isami, K., Nakamura, S., Shirakawa, H., Nakagawa, T., & Kaneko, S. (2012). Acute cold hypersensitivity characteristically induced by oxaliplatin is caused by the enhanced responsiveness of TRPA1 in mice. Molecular Pain, 8. https://doi.org/10.1186/1744-8069-8-55
