# Supplementary figures and images for: Pregabalin Silences Oxaliplatin-Activated Sensory Neurons to Relieve Cold Allodynia
Source: eNeuro. 2023 Feb 14;10(2):ENEURO.0395-22.2022. doi: 10.1523/ENEURO.0395-22.2022 (PMC9998121; doi:10.1523/ENEURO.0395-22.2022)

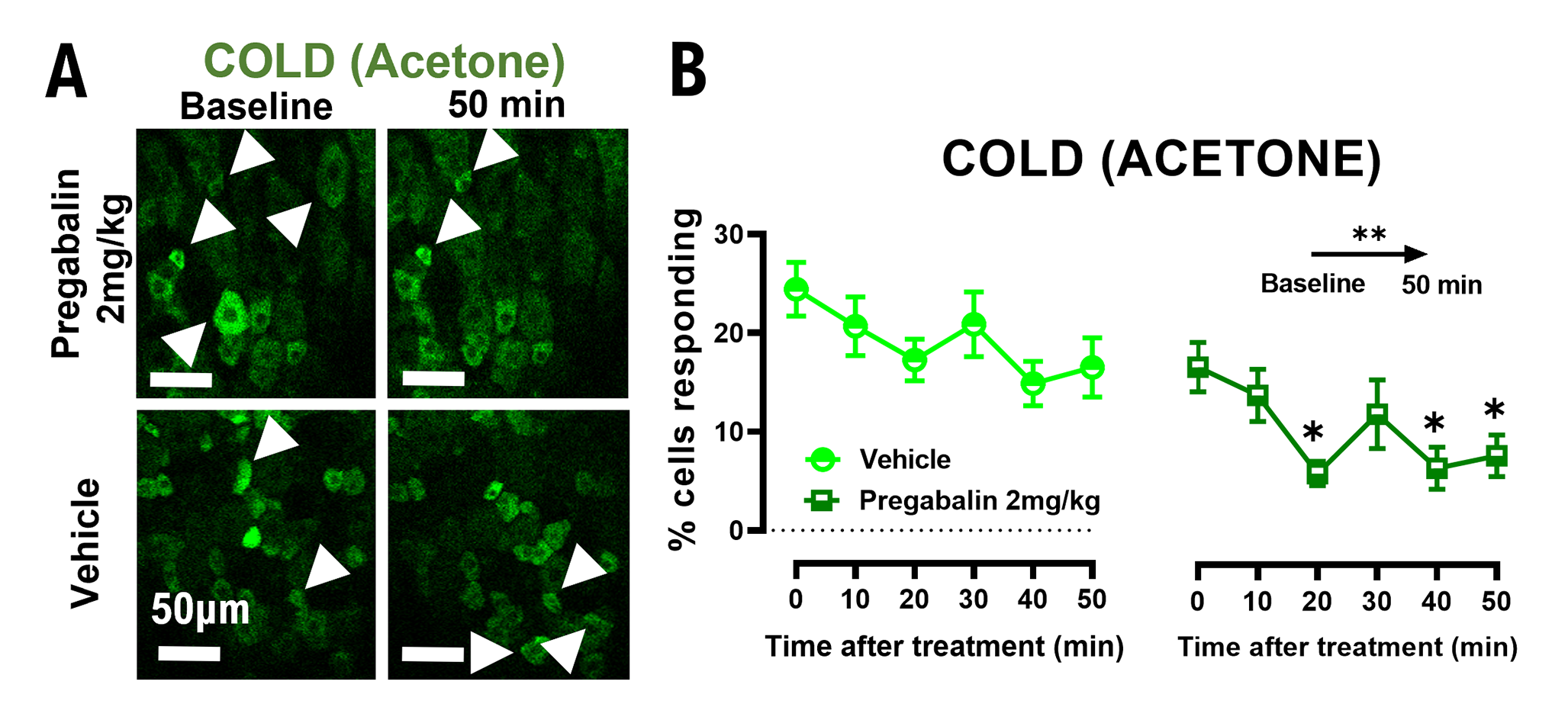

Supplement: Extended Data Figure 2-1 — Pregabalin treatment decreases the number of neurons responding to a chemical cold stimulus. A, Example images showing the reduction in the population of DRG neurons responding to acetone 50 min after treatment with 2 mg/kg of pregabalin. B, Graph showing the decrease of the percentages of cell responding to acetone. n = 6 pregabalin-treated mice, n = 5 vehicle-treated mice. Statistical analysis in B was performed using repeated measures ANOVA test with multiple comparisons. *p < 0.05, **p < 0.01. Download Figure 2-1, TIF file. [file enu-eN-NWR-0395-22-s03.tif]

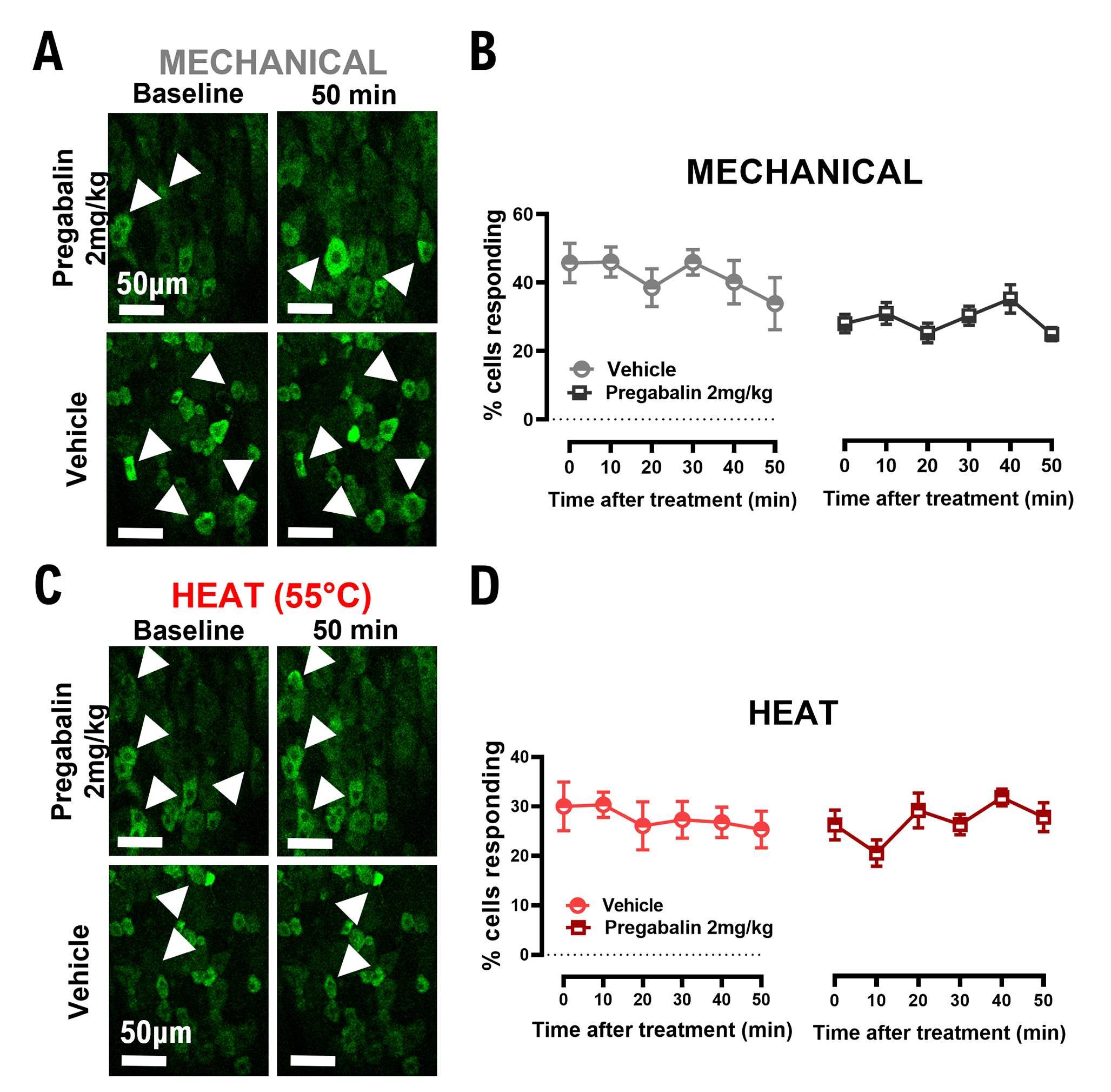

Supplement: Extended Data Figure 2-2 — Pregabalin treatment does not affect other sensory modalities besides cold. A, Example images showing no change in the population of DRG neurons responding to mechanical pinch 50 min after treatment with 2 mg/kg of pregabalin. B, Graph showing the unchanged percentages of cell responding to mechanical pinch. C, Example images showing no change in the population of DRG neurons responding to a 55°C water stimulus 50 min after treatment with 2 mg/kg of pregabalin. D, Graph showing the unchanged percentages of cell responding to a 55°C water stimulus. n = 6 pregabalin-treated mice, n = 5 vehicle-treated mice. Statistical analysis in B and D was performed using repeated measures ANOVA test with multiple comparisons. Download Figure 2-2, TIF file. [file enu-eN-NWR-0395-22-s04.tif]

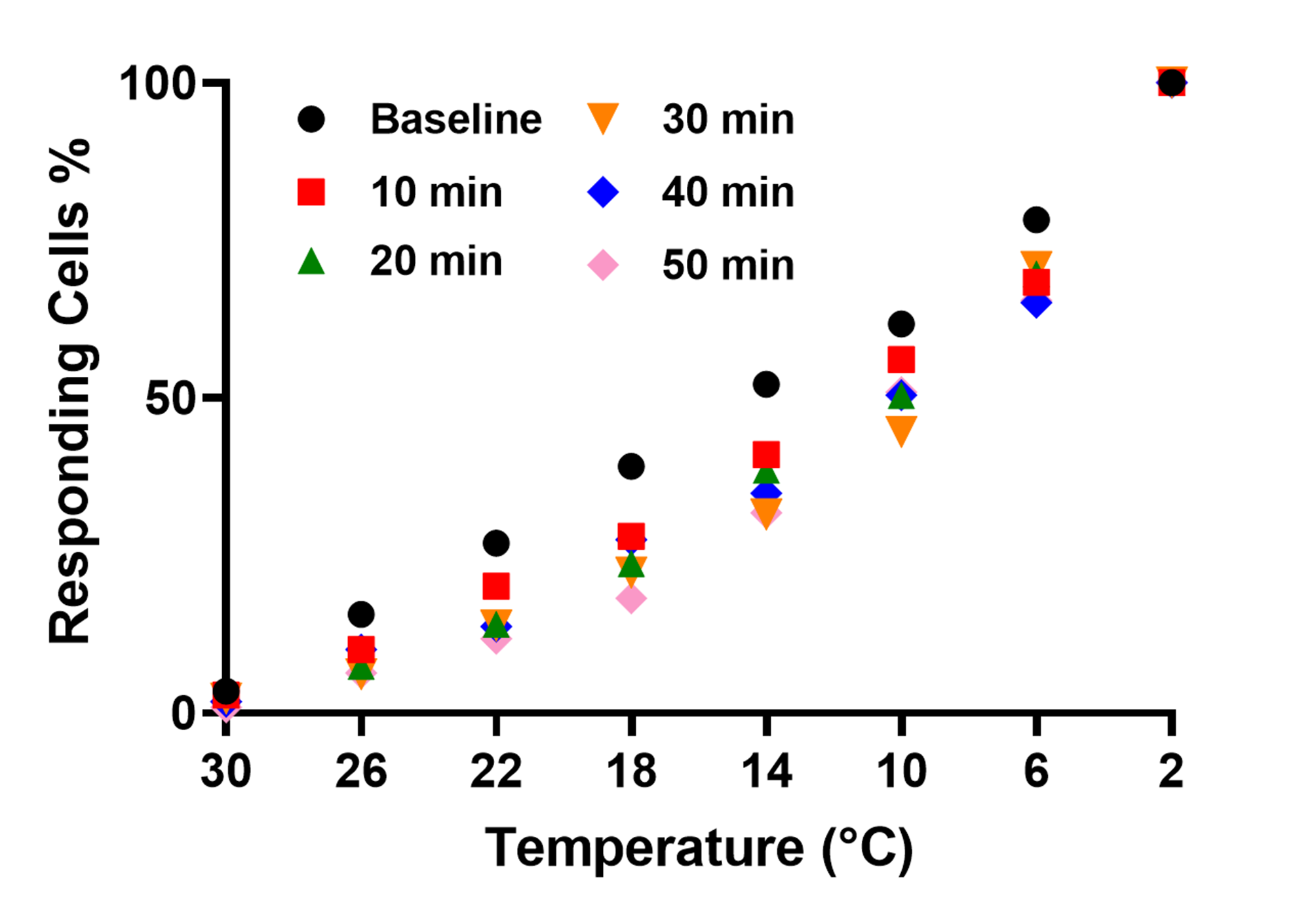

Supplement: Extended Data Figure 3-1 — Pregabalin treatment increases temperature threshold of cold-responding cells over time. Graph showing the change of the relationship between the number of cold-responding neurons and the temperature drop over time from pregabalin injection. Download Figure 3-1, TIF file. [file enu-eN-NWR-0395-22-s05.tif]

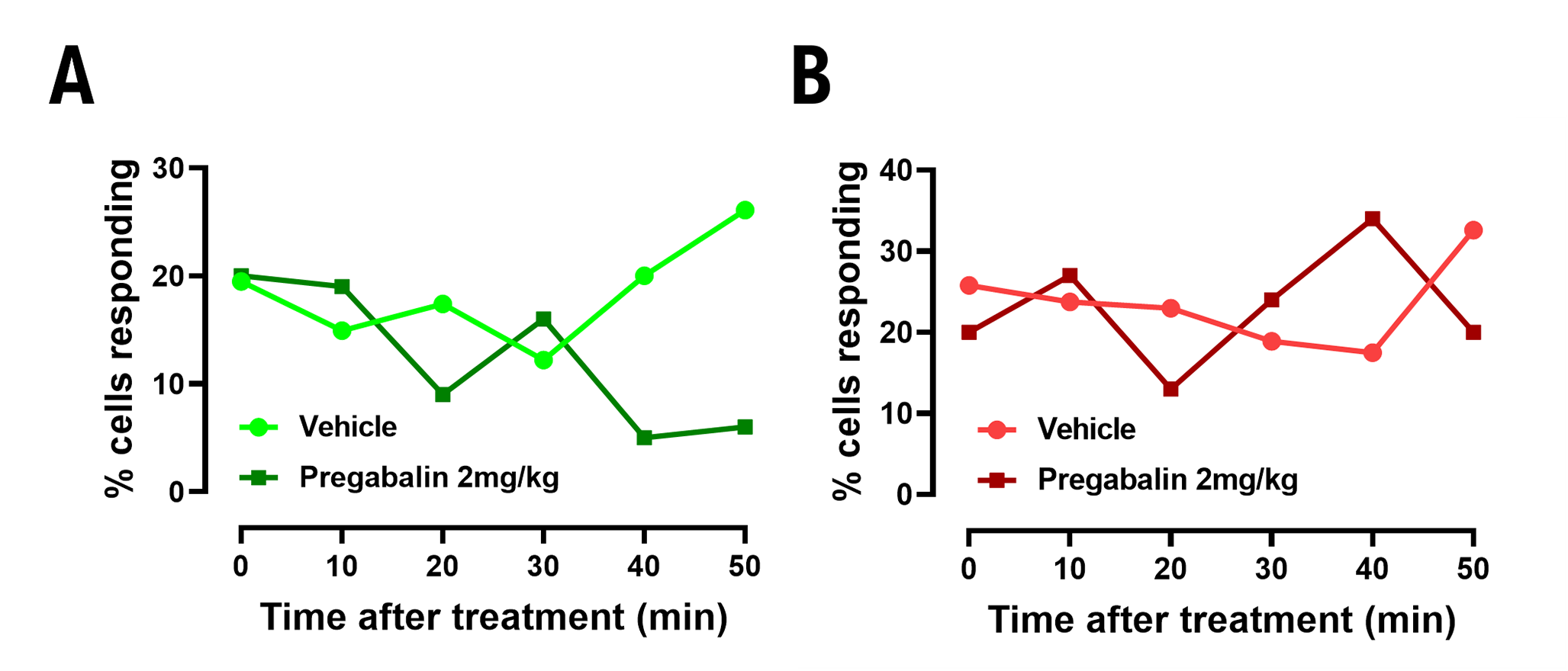

Supplement: Extended Data Figure 3-2 — Pregabalin treatment reduces the number of polymodal mechano-cold but not mechano-heat responding cells. A, Graph showing the change in the number of total mechano-responding neurons that respond also to acetone stimulus. Mice treated with vehicle do not exhibit the decrease over time from treatment as the mice treated with pregabalin do. B, Graph showing the change in the number of total mechano-responding neurons that respond also to a 55°C water stimulus. Mice do not exhibit significant differences in the percentage of polymodal mechano-heat responding neurons over time from pregabalin treatment. n = 6 pregabalin-treated mice, n = 5 vehicle-treated mice. Download Figure 3-2, TIF file. [file enu-eN-NWR-0395-22-s06.tif]

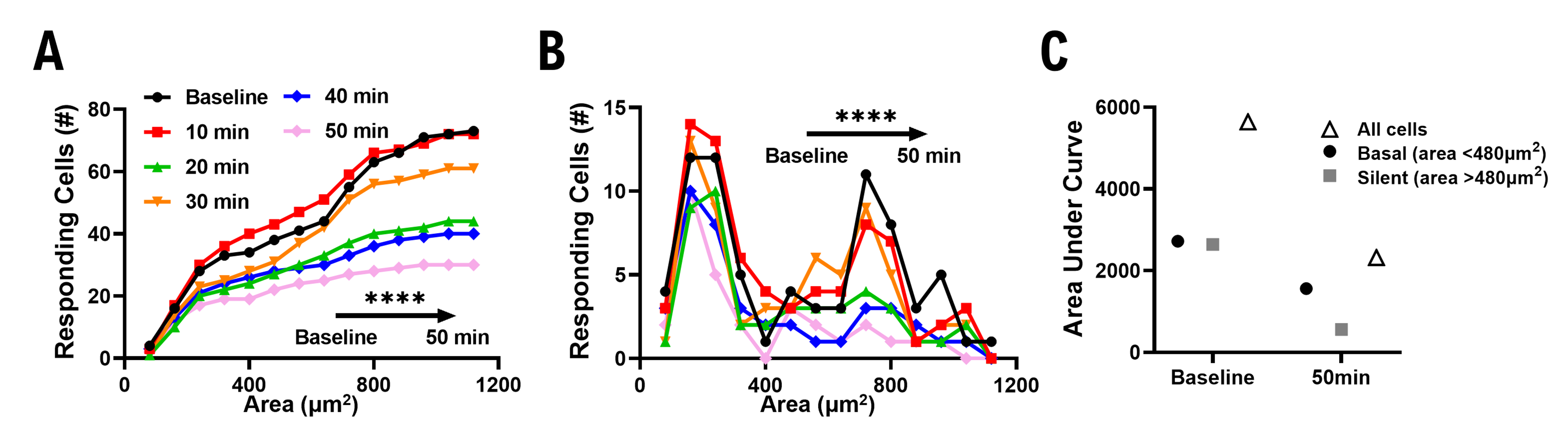

Supplement: Extended Data Figure 3-3 — Pregabalin preferentially inhibits the activity of “silent” cold-sensing neurons. A, Cumulative plots of the cell areas in oxaliplatin-treated mice before and after pregabalin injection. The difference between the distributions at different timepoints after treatment is statistically significant (p < 0.0001), as analyzed by Kruskal–Wallis test. B, Number plots of the distribution of cell areas of cold-responding cells in oxaliplatin-treated mice before and after pregabalin injection. The difference between the numeric distributions at different timepoints after treatment is statistically significant (p < 0.0001), as analyzed by Kruskal–Wallis test. C, Graph showing the area under curve changes before and 50 min after pregabalin injection. The cell areas are divided into basal cold responding cells (A < 480 μm2) and silent cold sensors (A > 480 μm2). Albeit there is a difference in both populations after 50 min from treatment, the population of silent cold sensors seems to be silenced almost completely. These thresholds have been calculated previously (MacDonald et al., 2021). Download Figure 3-3, TIF file. [file enu-eN-NWR-0395-22-s07.tif]

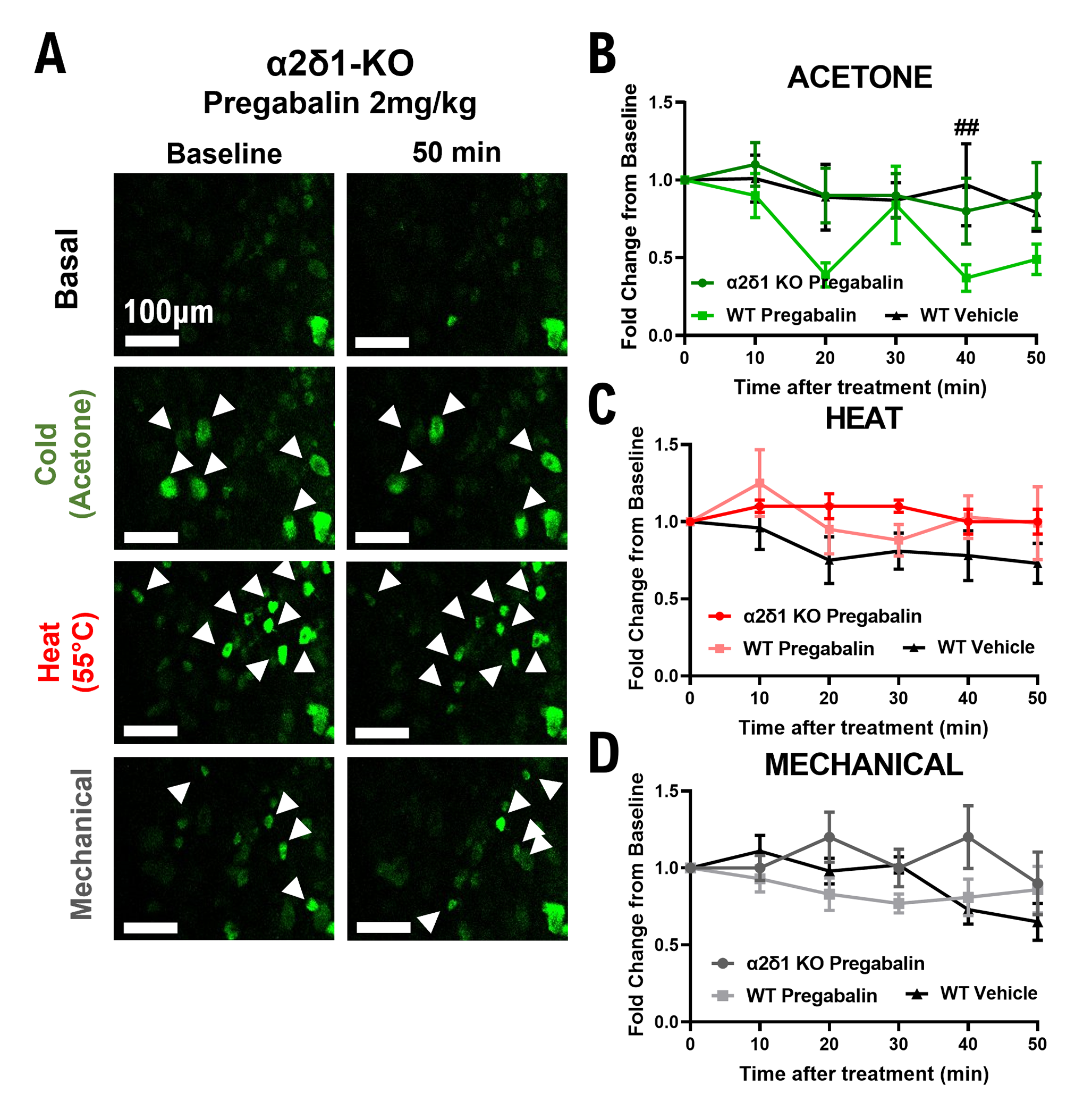

Supplement: Extended Data Figure 4-1 — Knock-out of the α2δ1 subunit of the VGCCs abolishes the effects of pregabalin on cold but has no effect on heat and mechanical responses. A, Example images showing no change in the population of DRG neurons responding to acetone, 55°C water, and mechanical pinch 50 min after treatment of α2δ1-KO mice with 2 mg/kg of pregabalin. B, Graph showing the unchanged percentages of cell responding to acetone in α2δ1-KO mice with respect to WT ones. C, Graph showing the unchanged percentages of cell responding to a 55°C water stimulus in α2δ1-KO mice with respect to WT ones. D, Graph showing the unchanged percentages of cell responding to mechanical pinch in α2δ1-KO mice with respect to WT ones. n = 7 α2δ1-KO mice, n = 6 pregabalin-treated mice, n = 5 vehicle-treated mice for imaging data. Statistical analyses in B–D were performed using repeated measures ANOVA test with multiple comparisons. ##p < 0.01 (α2δ1-KO vs WT pregabalin treated). Download Figure 4-1, TIF file. [file enu-eN-NWR-0395-22-s08.tif]
